# A Spleen‐Targeted Tolerogenic mRNA‐LNPs Vaccine for the Treatment of Experimental Asthma

**DOI:** 10.1002/advs.202412543

**Published:** 2025-02-08

**Authors:** Fazhan Wang, Jia Lou, Xiaohan Lou, Fang Wu, Xiaoke Gao, Xiaohan Yao, Jiajia Wan, Xixi Duan, Wenjing Deng, Lixia Ma, Lijing Zhang, Guangjie He, Ming Wang, Chen Ni, Ningjing Lei, Zhihai Qin

**Affiliations:** ^1^ Medical Research Center The First Affiliated Hospital of Zhengzhou University Zhengzhou University Zhengzhou Henan 450052 China; ^2^ Department of Pain and Rehabilitation Second Affiliated Hospital Army Medical University Chongqing 400038 China; ^3^ Department of Microbiology and Immunology School of Basic Medical Sciences Zhengzhou University Zhengzhou Henan 450001 China; ^4^ Department of Neuro‐Intensive Care Unit The First Affiliated Hospital of Zhengzhou University Zhengzhou Henan 450052 China; ^5^ Xinxiang Key Laboratory of Forensic Science Evidence School of Forensic Medicine Xinxiang Medical University Xinxiang Henan 453003 China

**Keywords:** celastrol, lipid nanoparticles, mRNA vaccine, spleen‐targeted delivery, tolerogenic immune responses

## Abstract

Lipid nanoparticles (LNPs)‐based mRNA vaccines have witnessed their great advantages in the fight against infectious diseases. However, the pro‐inflammatory properties of mRNA‐LNPs vaccines may hinder the induction of antigen‐specific tolerogenic immune responses. Here, it is demonstrated that stearic acid‐doped LNPs co‐loaded with nucleoside‐modified mRNA and celastrol selectively target spleen, convert their adjuvanticity and promote a tolerogenic rather than immunogenic DCs phenotype. Furthermore, the tolerogenic mRNA vaccine also invokes the generation of antigen‐specific regulatory T cells (Tregs) in the spleen and migration of the induced Tregs to the lung. In a mouse model of allergic asthma, immunization with the tolerogenic mRNA vaccine significantly alleviated symptom induction, reducing eosinophilic granulocyte accumulation and mucus secretion. In conclusion, this spleen‐targeted mRNA‐LNPs vaccine platform induces tolerogenic immune responses, offering promise for the development of therapeutics against allergic asthma and other conditions requiring immune tolerance modulation.

## Graphical abstract

Splenic DCs‐targeted co‐delivery of nucleoside‐modified antigen mRNA and Cel‐loaded LNPs vaccine induces tolerogenic DCs and subsequent antigen‐specific CD4^+^ regulatory T cells for the treatment of experimental allergic asthma.

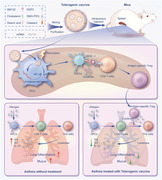

## 1 Introduction

Vaccines are widely recognized as highly effective means for preventing and treating various diseases.^[^ ^1^, ^2^, ^3^, ^4^, ^5^, ^6^, ^7^ ^]^ mRNA‐based vaccines, offering distinct advantages over peptide/protein or DNA‐based counterparts, have shown rapid advancement in recent years.^[^ ^8^, ^9^, ^10^, ^11^, ^12^ ^]^ Notably, mRNA‐lipid nanoparticles (LNPs) vaccines developed by Pfizer/BioNTech and Moderna have been swiftly approved for combating severe acute respiratory syndrome coronavirus 2 (SARS‐CoV‐2).^[^ ^13^ ^]^ In addition to eliciting antigen‐specific immunogenic responses against malignant tumors^[^ ^2^ ^]^ and infectious diseases,^[^ ^14^ ^]^ mRNA‐based vaccine approaches for preventing and treating allergic disorders^[^ ^15^ ^]^ and autoimmune diseases^[^ ^3^ ^]^ are rapidly evolving. The induction of tolerogenic immune responses is pivotal for managing conditions involving immune dysregulation such as autoimmunity, organ transplantation, chronic infections, and allergic diseases.^[^ ^16^, ^17^, ^18^ ^]^

Dendritic cells (DCs), as professional antigen‐presenting cells (APCs), can adopt either immunogenic^[^ ^2^ ^]^ or tolerogenic^[^ ^3^, ^19^ ^]^ characteristics based on their local environment, and play a pivotal role in initiating immune responses specific to antigens, either promoting immunity or inducing tolerance toward different immune cell types involved in immune dysfunction via contact‐dependent interactions and by secreting cytokines and metabolites.^[^ ^20^ ^]^ Endogenous stimuli such as interleukin (IL)‐10, and/or synthetic molecules such as rapamycin, induce a tolerogenic DCs phenotype, including changes in the expression of major histocompatibility complex (MHC) and co‐stimulatory molecules, production of cytokine and metabolites.^[^ ^20^ ^]^ Tolerogenic DCs can promote peripheral tolerance through enhancing the differentiation and expansion of regulatory T cells (Tregs), and/or facilitating the clonal anergy and deletion of self‐reactive CD8+ T cells, which has been investigated for the treatment of autoimmune diseases and other inflammatory disorders, such as rheumatoid arthritis, multiple sclerosis and transplantation.^[^ ^21^ ^]^ However, a standardized method to generate tolerogenic DCs ex vivo remains to be developed. Thus, the generation of tolerogenic DCs in vivo represents a promising alternative for tolerogenic DCs‐based therapies.^[^ ^22^ ^]^ However, the inflammatory nature of mRNA‐LNPs vaccines might hinder the development of tolerogenic DCs. Modifying the mRNA with nucleoside adjustments can reduce immune recognition and enhance protein production efficiency.^[^ ^3^, ^23^ ^]^ Despite these modifications, LNPs used for mRNA delivery remain highly inflammatory due to their ionizable lipid component.^[^ ^24^ ^]^ The inclusion of bioactive molecules with potent anti‐inflammatory activity may convert the immunogenic adjuvanticity of LNPs to tolerogenic adjuvanticity for disease immunotherapy.^[^ ^25^ ^]^ Celastrol (Cel), derived from Tripterygium wilfordii, has potent anti‐inflammatory and immune‐suppressive properties.^[^ ^26^, ^27^ ^]^ Therefore, the uptake of Cel‐loaded LNPs may inhibit the maturation of DCs induced by inflammatory LNPs, maintaining them in a tolerogenic and non‐activated state.

The spleen, as the largest secondary lymphoid organ, regulates immune responses both locally and systemically.^[^ ^28^, ^29^, ^30^ ^]^ Its unique physical structure facilitates infrequent interactions between APCs and specific lymphocytes, making it an ideal target for vaccines.^[^ ^31^, ^32^, ^33^ ^]^ Previously, we have constructed a splenic DCs‐targeted mRNA‐LNPs vaccine platform through doping natural long‐chain saturated fatty acids in the formulation of lipid component,^[^ ^34^ ^]^ investigated its protective immune response against various tumors in mice models^[^ ^32^ ^]^ and discussed the application of spleen‐targeting nanosystems for immunomodulation.^[^ ^35^ ^]^ As an allergic inflammatory disease, asthma is characterized by allergic inflammation, involves activation of the T helper 2 (Th2)‐cell pathway triggered by allergen uptake by DCs.^[^ ^36^, ^37^ ^]^ Currently, the general treatments for asthma could only relieve asthmatic symptoms but hardly prevent the deteriorative progression of the disease, and each has limitations. Antigen‐specific tolerance‐inducing immunotherapy has shown promise in treating conditions like arthritis,^[^ ^19^, ^38^ ^]^ experimental autoimmune encephalomyelitis,^[^ ^3^, ^39^, ^40^ ^]^ and peanut‐induced anaphylaxis^[^ ^15^ ^]^ and represents a potentially curative method for asthma treatment.

**Figure 1:**
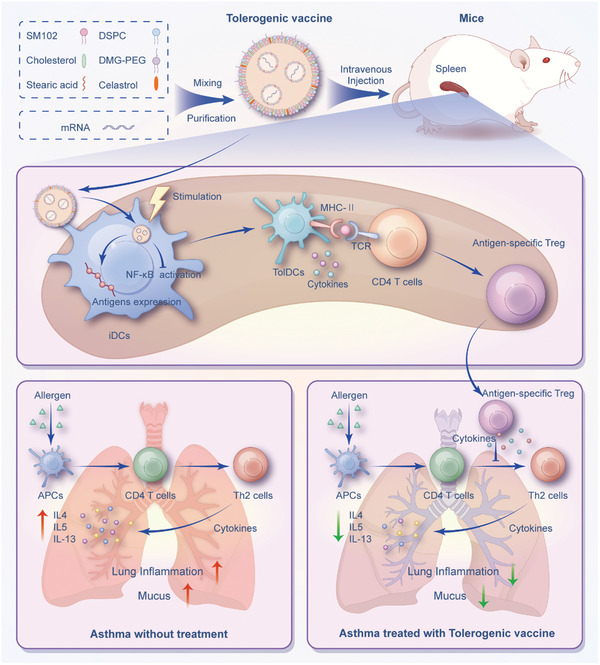
Schematic illustration of splenic DCs‐targeted tolerogenic mRNA‐LNPs vaccine against experimental asthma. Splenic DCs‐targeted co‐delivery of nucleoside‐modified antigen mRNA and Cel‐loaded LNPs vaccine for the treatment of experimental allergic asthma through inducing tolerogenic DCs and subsequent antigen‐specific CD4^+^ regulatory T cells (Tregs) mediated immunological tolerance. iDCs, immature dendritic cells; tolDCs, tolerogenic dendritic cells; Tregs, regulatory T cells; APCs, antigen‐presenting cells.

In this study, we investigated the feasibility of delivering nucleoside‐modified antigen mRNA and celastrol‐loaded LNPs specifically to splenic DCs with the aim of inducing tolerogenic splenic DCs and subsequently promoting allergen‐specific Tregs for the treatment of allergic asthma (**Figure** **1**). The potent immunotherapeutic efficacy of our spleen‐targeted mRNA‐LNPs vaccine against experimental asthma by induction of tolerogenic immune response may pave the way for future clinical translation of tolerogenic mRNA‐LNPs vaccine in treating allergies such as asthma.

## 2 Results

### 2.1 Characterization of Spleen‐Targeted Tolerogenic mRNA‐LNPs Vaccine Platform

**Figure 2:**
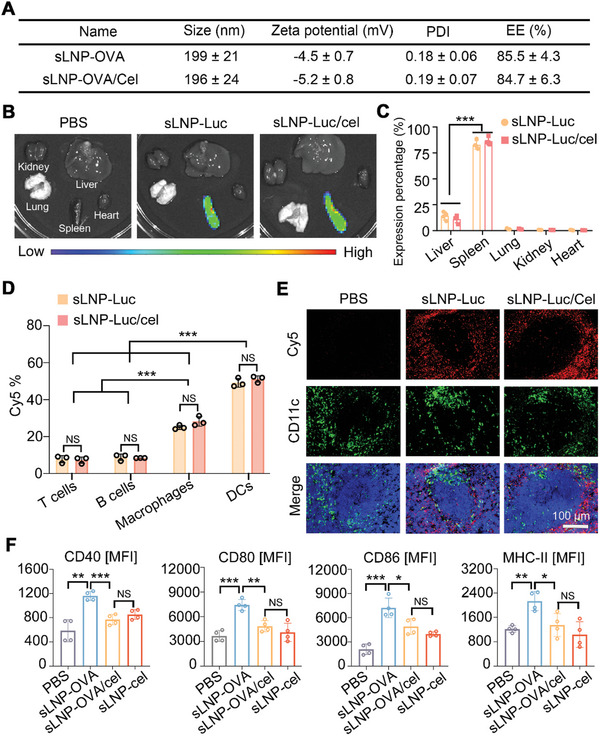
Characterization of spleen‐targeted LNPs‐based tolerogenic mRNA vaccine delivery systems. A) Particle sizes, zeta potential, polydispersity index (PDI), and mRNA encapsulation efficiency (EE) of stearic acid‐doped mRNA‐LNPs vaccine delivery systems with or without celastrol inclusion were measured. Error bars represent mean ± SD (n = 3 independent samples). B) Representative bioluminescence imaging of organs isolated from mice 6 h after intravenous injection of the mRNA‐LNPs vaccine platform. C) Quantification of transfection activity of the mRNA‐LNPs vaccine delivery system in the liver, spleen, lung, kidney, and heart. Error bars represent mean ± SEM (n = 3 biologically independent samples). D) Flow cytometry analysis of the percentages of Cy5 in T cells, B cells, macrophages, and DCs. Error bars represent mean ± SEM (n = 3 biologically independent samples). E) Immunostaining of DCs in the spleen 6 h after intravenous injection of Cy5‐labeled sLNP‐OVA/Cel, demonstrating co‐localization of DCs and sLNP‐OVA/Cel in the spleen. Scale bars: 100 µm. F) Quantitative analysis of the costimulatory molecules CD40, CD80, CD86, and MHC‐II in splenic DCs. Error bars represent mean ± SEM (n = 4 biologically independent samples). Statistical comparisons were performed using one‐way ANOVA with Tukey's test. NS, no significance; *P < 0.05; **P < 0.01; ***P < 0.001.

The tolerogenic mRNA‐LNPs vaccine platform was formulated by mixing an ethanol phase containing SM102, DSPC, Chol, DMG‐PEG, stearic acid and celastrol with an aqueous phase containing nucleoside‐modified mRNA. We first evaluated the impact of celastrol inclusion on the physicochemical properties of the stearic acid‐doped LNPs loaded with ovalbumin (OVA) mRNA (sLNP‐OVA). Dynamic light scattering (DLS) analysis revealed that the addition of celastrol (sLNP‐OVA/Cel) did not significantly alter the size (199 ± 21 nm versus 196 ± 24 nm) or zeta potential (−4.5 ± 0.7 mV vs −5.2 ± 0.8 mV) of the mRNA‐LNPs vaccine platform (**Figure** **2**A). Moreover, there were no significant differences in mRNA encapsulation efficiency between sLNPs‐OVA (85.5 ± 4.3%) and sLNPs‐OVA/Cel (84.7 ± 6.3%) (Figure 2A). Agarose gel assays confirmed nearly complete mRNA entrapment within OVA‐mRNA‐loaded sLNPs, regardless of celastrol inclusion (Figure S1A, Supporting Information). Transmission electron microscopy (TEM) images further demonstrated homogeneous spherical nanoparticles in both sLNPs‐OVA and sLNPs‐OVA/Cel formulations (Figure S1B, Supporting Information). Next, we investigated the impact of celastrol on mRNA translation efficiency using the tolerogenic mRNA‐LNPs vaccine platform, substituting OVA‐mRNA with luciferase mRNA (Luc‐mRNA). Imaging analysis confirmed luciferase protein expression exclusively in the spleen (Figure 2B). Quantitatively, there was no significant difference in luciferase protein expression between spleens treated with mRNA‐LNPs vaccines with or without celastrol (Figure 2C).

To examine the splenic DCs selectivity of the tolerogenic mRNA‐LNPs vaccine after intravenous injection, we synthesized mRNA‐LNP delivery systems with or without celastrol, substituting 33.3% of the cholesterol content with Cy5‐labeled cholesterol. These particles were administered intravenously to BALB/c mice at 0.5 mg kg^−1^ (n = 3), followed by the collection of IVIS images and flow cytometry analysis 6 h later to determine the in vivo biodistribution profiles of the mRNA‐LNPs vaccine platform. The results showed no significant differences in the accumulation of mRNA‐LNPs delivery systems with or without celastrol in the major organs (Figure S1C, Supporting Information). Part of the spleen was submitted to flow cytometry, and the remaining part underwent tissue sectioning. Flow cytometry confirmed the cellular uptake of the tolerogenic mRNA‐LNPs vaccine platform in the spleen 6 h post‐injection (Figure S1D, Supporting Information). Approximately 51.13 ± 1.78% of splenic DCs were Cy5‐positive, which was significantly higher than Cy5‐positive B cells (8.42 ± 0.03%), T cells (7.29 ± 1.71%), and macrophages (28.27 ± 2.61%) (Figure 2D), indicating the excellent splenic DCs selectivity of the stearic acid‐doped LNPs platform. Importantly, the inclusion of celastrol did not affect the uptake of stearic acid‐doped LNP‐based mRNA delivery systems by splenic cells (Figure 2D). After spleen tissue sectioning, digital fluorescence microscopy was performed using FITC‐conjugated anti‐CD11c antibody staining. Confocal laser‐scanning microscopy revealed limited distribution of the mRNA‐LNPs vaccine platform with celastrol in the marginal zone around the white pulp, with co‐localization of mRNA‐LNPs and splenic DCs, similar to that observed with mRNA‐LNPs vaccine platform without celastrol (Figure 2E).

To further explore the reduction in immunogenicity conferred by celastrol inclusion, we developed an OVA‐mRNA‐loaded stearic acid‐doped mRNA‐LNPs vaccine platform with or without celastrol. Splenocyte suspensions were prepared 24 h post‐injection and stained with antibodies against CD45, CD11c, CD40, CD80, CD86, MHC‐II, CD4, CD8, B220, and CD69 for analysis. Splenocytes from mice treated with sLNP‐OVA/Cel or sLNP/Cel showed lower expression levels of CD40, CD80, CD86, and MHC‐II on splenic DCs compared to those treated with sLNP‐OVA (Figure 2F). Additionally, we evaluated the activation state of splenic B cells, CD4^+^ T cells, and CD8^+^ T cells by gating CD45^+^ B220^+^ cells, CD45^+^ CD4^+^ cells or CD45^+^CD8^+^ cells, respectively (Figure S2A, Supporting Information). Compared to mice injected with sLNP‐OVA, the expression of CD69 on splenic B cells, CD4^+^ T cells and CD8^+^ T cells was also reduced in mice treated with sLNP‐OVA/Cel or sLNP/Cel (Figure S2B,C, Supporting Information). These findings suggest that the inclusion of celastrol restricts the activation splenic immune cells following mRNA‐LNPs delivery systems intravenous injection in vivo.

### 2.2 Tolerogenic mRNA‐LNPs Vaccine Promotes Tolerogenic DCs Differentiation In Vitro

**Figure 3:**
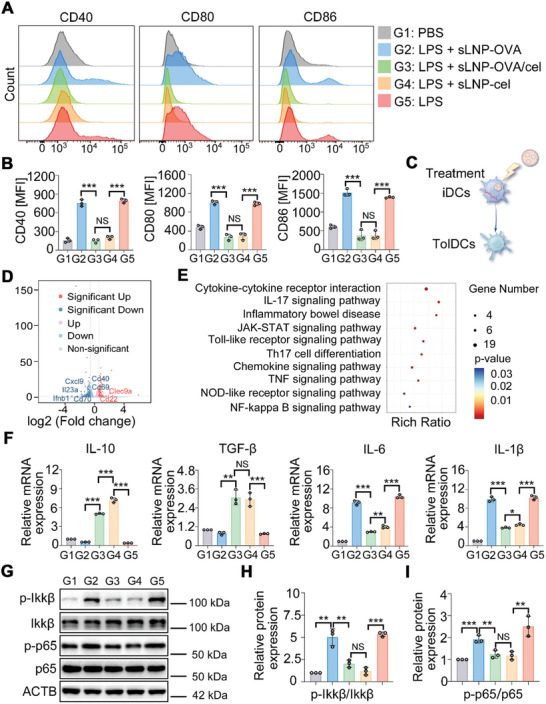
sLNP‐OVA/Cel‐mediated the induction of tolerogenic DCs in vitro. A) Representative histograms showing CD40, CD80, and CD86 expression in BMDCs treated with different formulations treatments followed by stimulation with LPS for 24 h. PBS‐treated cells were negative control, whereas cells treated with LPS alone were positive control. B) Quantitative analysis of the costimulatory molecules CD40, CD80, and CD86 in BMDCs. Error bars represent mean ± SEM (n = 3 biologically independent samples). C) Induction scheme of the generation of tolerogenic DCs. Immature BMDCs were treated with sLNPs‐based mRNA vaccine delivery systems loaded with or without celastrol, followed by LPS stimulation. D) Volcano plots depicting differentially expressed genes (DEGs) in BMDCs treated with sLNPs‐OVA or sLNPs‐OVA/Cel. E) Gene Ontology (GO) enrichment analysis of signaling pathways in BMDCs treated with sLNPs‐OVA or sLNPs‐OVA/Cel. F) Relative mRNA levels of anti‐inflammatory cytokines IL‐10 and TGF‐β, and pro‐inflammatory cytokines IL‐6 and IL‐1β in BMDCs. Error bars represent mean ± SEM (n = 3 biologically independent samples). G) Representative Western blot images of p65, p‐p65, lkkβ and p‐lkkβ on BMDCs treated with different formulations. H) Quantitative analysis of relative expression of p‐Ikkβ. Error bars represent mean ± SEM (n = 3 biologically independent samples). I) Quantitative analysis of relative expression of p‐p65. Error bars represent mean ± SEM (n = 3 biologically independent samples). iDCs, immature dendritic cells; tolDCs, tolerogenic dendritic cells. Statistical comparisons were performed using one‐way ANOVA with Tukey's test. NS, no significance; *P < 0.05; **P < 0.01; ***P < 0.001.

To investigate whether our particle design promotes the differentiation of tolerogenic rather than inflammatory DCs from immature DCs, we examined the anti‐maturation effect of celastrol‐loaded nucleoside‐modified mRNA‐LNPs delivery systems on bone marrow‐derived dendritic cells (BMDCs). First, we assessed whether the inclusion of celastrol affected the cellular uptake of mRNA‐LNPs delivery systems in vitro. Confocal imaging and flow cytometry were performed on BMDCs incubated with sLNP‐OVA or sLNP‐OVA/Cel. Confocal images showed that BMDCs efficiently internalized the vaccine delivery systems regardless of celastrol addition (Figure S3A, Supporting Information). Furthermore, flow cytometry confirmed similar cellular uptake percentages between sLNP‐OVA/Cel (95.25 ± 2.32%) and sLNP‐OVA (96.18 ± 1.86%) (Figure S3B, Supporting Information). Next, BMDCs were cultured with different mRNA‐LNPs vaccine platforms for 4 h and then activated with lipopolysaccharide (LPS) for 24 h to induce maturation. Flow cytometry analysis revealed that LPS‐treated BMDCs (mature DCs, mDCs) significantly upregulated the expression of co‐stimulatory molecules (CD40, CD80, CD86) compared to PBS‐treated BMDCs (immature DCs, iDCs) (**Figure** **3**A,B). In contrast, BMDCs treated with sLNP‐OVA/Cel or sLNP‐Cel (tolerogenic DCs) showed lower levels of these co‐stimulatory molecules similar to iDCs, significantly different from mDCs and sLNP‐OVA‐treated BMDCs (Figure 3A,B). Thus, celastrol and mRNA co‐loaded vaccine induced a tolerogenic rather than immunogenic phenotype in immature BMDCs following LPS stimulation (Figure 3C). Furthermore, RNA‐seq analysis identified over 194 differentially expressed genes (DEGs) associated with sLNPs‐OVA/Cel or sLNPs‐OVA treatment (Figure 3D). Kyoto Encyclopedia of Genes and Genomes (KEGG) enrichment analysis revealed enrichment of DEGs in immune‐stimulating pathways such as Cytokine, Chemokine, and NF‐κB signaling pathways with different treatments (Figure 3E). In addition, the key genes involved in pro‐inflammatory and anti‐inflammatory cytokine production were examined. According to the results of quantitative reverse transcription polymerase chain reaction (qRT‐PCR), higher mRNA levels of anti‐inflammatory cytokines (TGF‐β and IL‐10) and lower levels of inflammatory cytokines (IL‐6 and IL‐1β) in sLNP‐OVA/Cel‐treated BMDCs were observed compared to sLNP‐OVA‐treated BMDCs (Figure 3F). Furthermore, flow cytometry (Figure S3C,D, Supporting Information) and enzyme‐linked immunosorbent assay (ELISA) (Figure S3E, Supporting Information) confirmed higher levels of anti‐inflammatory cytokines (TGF‐β and IL‐10) in sLNP‐OVA/Cel‐treated BMDCs compared to sLNP‐OVA‐treated BMDCs. Moreover, the activation state of the NF‐κB signaling pathway in BMDCs was significantly enhanced after stimulation with LPS (Figure 3G). However, when the BMDCs were pretreated with the sLNP‐OVA/Cel, the NF‐κB signaling pathway was significantly suppressed compared to that of BMDC pretreated with sLNP‐OVA or treated solely with LPS, as demonstrated by the significantly decreased phosphorylation of P65 and Ikkβ (Figure 3G–I). Collectively, these data demonstrate that the inclusion of celastrol in nucleoside‐modified mRNA‐LNPs delivery systems can induce tolerogenic DCs differentiation by suppressing the NF‐κB signaling pathway.

### 2.3 Generation and Migration of Functional Tregs In Vitro and In Vivo

**Figure 4:**
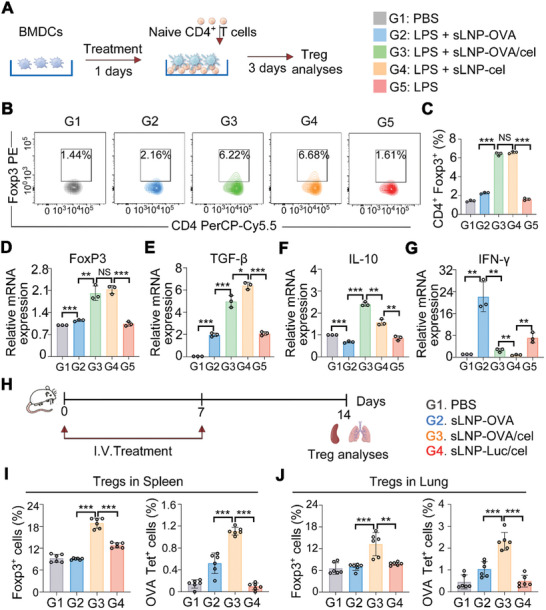
Generation and migration of functional Tregs by tolerogenic mRNA‐LNPs vaccine. A) Schematic representation of BMDCs treatment with tolerogenic mRNA vaccine, induction of Tregs, and subsequent Treg analyses. B) Flow cytometric analysis showing induction of Tregs by BMDCs treated with different nanovaccines. C) Quantitative analysis of the proportion of Tregs in CD4^+^ T cells. Error bars represent mean ± SEM (n = 3 biologically independent samples). D–G) Relative mRNA expression levels of Foxp3 (D), TGF‐β (E), IL‐10 (F), and IFN‐γ (G) in CD4^+^ T cells co‐cultured with BMDCs treated with different nanovaccines. Error bars represent mean ± SEM (n = 3 biologically independent samples). H) Schematic diagram illustrating the immunization process, sample collection, and subsequent Treg analyses. I) Flow cytometric analysis showing induction of splenic Tregs and antigen‐specific Tregs in situ by the tolerogenic mRNA vaccine. Error bars represent mean ± SEM (n = 6 biologically independent samples). J) Flow cytometric analysis demonstrating accumulation of Tregs and antigen‐specific Tregs in the lung. Error bars represent mean ± SEM (n = 6 biologically independent samples). Statistical comparisons were performed using one‐way ANOVA with Tukey's test. NS, no significance; *P < 0.05; **P < 0.01; ***P < 0.001.

The generation of tolerogenic DCs by the tolerogenic mRNA vaccine platform prompted us to investigate whether sLNP‐OVA/Cel could induce functional Tregs both in vitro and in vivo. To assess the ability of celastrol addition in the mRNA‐LNPs vaccine platform to promote functional Treg generation in vitro, splenocyte suspensions were used to isolate CD4^+^ T cells by magnetic beads before co‐culturing with differently treated BMDCs for 72 h (**Figure** **4**A). Flow cytometry analysis of non‐adherent cells stained with anti‐mouse CD4 and anti‐mouse Forkhead box P3 (Foxp3) antibodies showed that BMDCs treated with sLNP‐OVA/Cel induced a significantly higher proportion of Tregs compared to sLNP‐OVA treated BMDCs (Figure 4B,C; Figure S4A, Supporting Information). Additionally, mRNA expression analysis revealed higher levels of the Treg‐specific transcription factor Foxp3, as well as the immunosuppressive cytokines IL‐10 and TGF‐β, in the sLNP‐OVA/Cel treated group, while inflammatory cytokine IFN‐γ levels were lower compared to sLNP‐OVA treated BMDCs (Figure 4D–G). Moreover, the IL‐10 and TGF‐β‐producing Treg clusters were further evaluated. The sLNP‐OVA/Cel group showed significantly higher levels of IL‐10 and TGF‐β in Tregs than the other groups (Figure S4B,C, Supporting Information). Functional assays demonstrated that Tregs generated from sLNP‐OVA/Cel treated BMDCs effectively inhibited the differentiation of naive CD4^+^ T cells into Th2 cells compared to those generated from sLNP‐OVA treated BMDCs (Figure S4D,E, Supporting Information). These findings highlight the efficacy of the tolerogenic mRNA vaccine platform in inducing tolerogenic DCs and promoting the differentiation of functional Tregs in vitro.

To assess the generation of Tregs and antigen‐specific Tregs in vivo, the animals were intravenously injected with 0.5 mg kg^−1^ of the tolerogenic mRNA vaccine once a week for two weeks. Seven days after the final immunization, splenocyte and lung cell suspensions were prepared for analysis (Figure 4H). Flow cytometry analysis revealed a significant increase in the percentage of splenic Tregs within B220^−^CD4^+^ T cells and OVA‐specific tetramer‐positive splenic CD4^+^ Foxp3^+^ Tregs in mice treated with sLNP‐OVA/Cel compared to untreated or sLNP‐OVA treated mice (Figure 4I). Moreover, sLNP‐OVA/Cel treatment resulted in a significant decrease in effector memory T cells compared to sLNP‐OVA treated mice, suggesting potential long‐term immune tolerance (Figure S5A,B, Supporting Information).

To further investigate whether splenic Tregs could migrate to the lung and exert immunosuppressive functions, lung cell suspensions were analyzed by flow cytometry. The results showed that the percentages of Tregs within lung CD4^+^ T cells and OVA‐specific tetramer‐positive lung Tregs were significantly higher in the sLNP‐OVA/Cel treated group compared to PBS or sLNP‐OVA treated groups (Figure 4J). These results demonstrate that intravenous administration of sLNP‐OVA/Cel induced significant generation of OVA‐specific splenic Tregs in situ, and subsequently migrated to the lung to exert immunosuppressive functions.

### 2.4 Prophylactic Effects of Tolerogenic mRNA‐LNPs Vaccine Against Experimental Asthma

**Figure 5:**
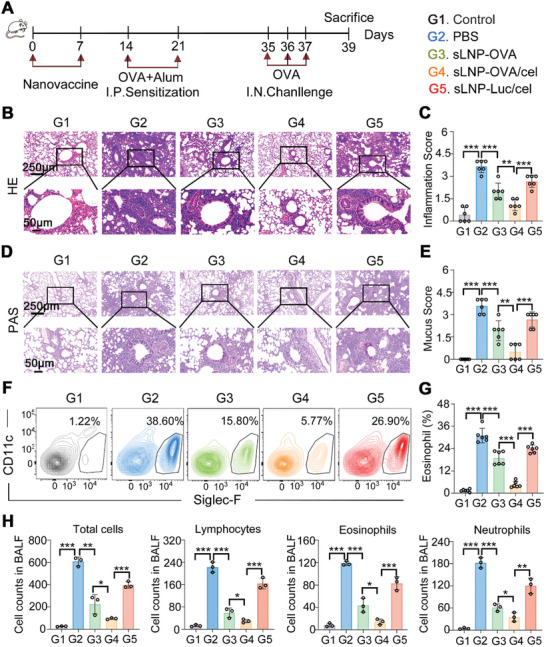
Prophylactic effects of sLNP‐OVA/Cel against experimental allergic asthma. A) Schematic diagram illustrating the protocol for intravenous immunization, intraperitoneal sensitization, and intratracheal challenge for preventive immunotherapy against allergic asthma. B) Representative H&E staining images of lungs from PBS and nanovaccine‐treated asthma mice at the end of the study. Scale bars: 250 µm (top row) and 50 µm (bottom row). C) Quantitative analysis of peribronchial inflammation in Control and nanovaccine‐treated asthma mice at the end of the study. Error bars represent mean ± SEM (n = 6 biologically independent samples). D) Representative PAS staining images of lungs from Control and nanovaccine‐treated asthma mice at the end of the study. Scale bars: 250 µm (top row) and 50 µm (bottom row). E) Quantitative analysis of peribronchial mucus in Control and nanovaccine‐treated asthma mice at the end of the study. Error bars represent mean ± SEM (n = 6 biologically independent samples). F) Flow cytometric analysis of eosinophil infiltration in lungs from Control and nanovaccine‐treated asthma mice. G) Quantitative analysis of eosinophil infiltration in lungs from Control and nanovaccine‐treated asthma mice. Error bars represent mean ± SEM (n = 6 biologically independent samples). H) Total cell numbers in BALF and numbers of lymphocytes, eosinophils, and neutrophils. Error bars represent mean ± SEM (n = 3 biologically independent samples). Statistical comparisons were performed using one‐way ANOVA with Tukey's test. *P < 0.05; **P < 0.01; ***P < 0.001.

To evaluate the prophylactic efficacy of the tolerogenic mRNA vaccine, BALB/c mice were given two intravenous injections of 0.5 mg kg^−1^ sLNP‐OVA, sLNP‐OVA/Cel, or sLNP‐Luc/Cel once a week. The animals were sensitized intraperitoneally with OVA twice a week for two weeks, followed by an intratracheal challenge to induce experimental allergic asthma models (**Figure** **5**A). At the end of the prophylactic study, half of each lung was fixed in 4% formaldehyde and sectioned for subsequent experiments. Hematoxylin and eosin (H&E) staining revealed that asthma induction led to lung inflammation, with sLNP‐OVA/Cel injection showing more effective inhibition of lung inflammation compared to other treatments (Figure 5B). The inflammation score of the sLNP‐OVA group was significantly lower than that of the PBS group (Figure 5C). Importantly, the inflammation score of the sLNP‐OVA/Cel group was significantly lower than that of the sLNP‐OVA group (Figure 5C). Mucus production in lung tissues, assessed by periodic acid‐Schiff (PAS) staining and scored accordingly, was reduced in the sLNP‐OVA/Cel treated group compared to PBS, sLNP‐OVA, or sLNP‐Luc/Cel treated groups (Figure 5D,E). Pulmonary cell suspensions were also analyzed. Notably, sLNP‐OVA treatment significantly reduced lung neutrophil infiltration compared to PBS‐treated asthma mice (Figure 5F,G), while sLNP‐OVA/Cel treatment resulted in even less neutrophil infiltration compared to sLNP‐OVA‐treated mice (Figure 5F,G). Additionally, analysis of bronchoalveolar lavage fluid (BALF) cell counts showed that total immune cells, lymphocytes, eosinophils, and neutrophils were lowest in the tolerogenic vaccine group compared to other treatment groups (Figure S5C,H, Supporting Information). Furthermore, levels of OVA‐specific IgE and Th2‐associated cytokines (IL‐4, IL‐5, IL‐13) in BALF were significantly reduced in the sLNP‐OVA/Cel treated group compared to other asthma treatment groups (Figure S5D,E, Supporting Information). These findings confirm the potent prophylactic immunotherapy efficacy of the tolerogenic sLNP‐OVA/Cel vaccine against experimental allergic asthma.

### 2.5 Immunomodulatory Effect of Tolerogenic mRNA‐LNPs Vaccine in the Lung and Spleen of Asthma Mice

**Figure 6:**
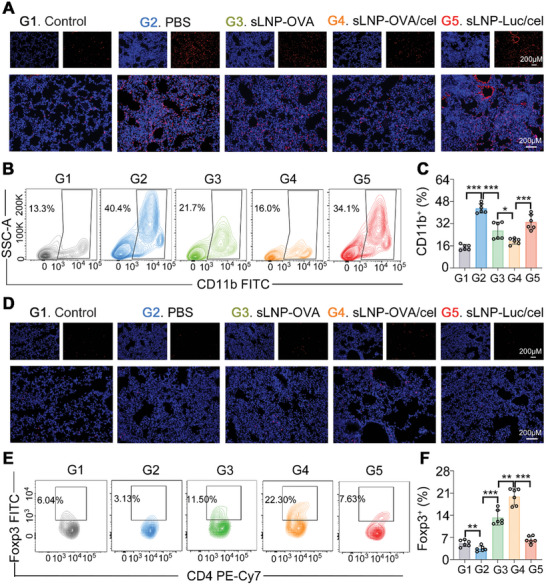
In Immunomodulatory effect of sLNP‐OVA/Cel in the lung of asthma mice at the end of the study. A) Representative immunofluorescence staining images of lungs with anti‐CD11b antibody from PBS and nanovaccine‐treated asthma mice. Scale bars: 200 µm (top row) and 200 µm (bottom row). B,C) Quantitative analysis of inflammatory monocyte infiltration in the lung by flow cytometry. Error bars represent mean ± SEM (n = 6 biologically independent samples). D) Representative immunofluorescence staining images of lungs with anti‐Foxp3 antibody from Control and nanovaccine‐treated asthma mice. Scale bars: 200 µm (top row) and 200 µm (bottom row). E,F) Quantitative analysis of Treg infiltration in the lung by flow cytometry. Error bars represent mean ± SEM (n = 6 biologically independent samples). Statistical comparisons were performed using one‐way ANOVA with Tukey's test. *P < 0.05; **P < 0.01; ***P < 0.001.

The infiltration of immune cells into the lungs plays an essential role in the progression of inflammatory asthma. Here, we investigated whether sLNP‐OVA/Cel injection could mitigate asthma by modulating the infiltration of inflammatory and suppressive immune cells in the lung through immunostaining analyses of lung sections (**Figure** **6**A,D) and flow cytometry of lung cell suspensions (Figure 6B,C,E,F). The results showed that sLNP‐OVA/Cel immunization significantly suppressed the infiltration of inflammatory monocytes in the lung compared to Control groups (Figure 6A–C). Additionally, the accumulation of CD45^+^ immune cells and CD4^+^ T cells was markedly reduced in mice treated with sLNP‐OVA/Cel compared to those treated with sLNP‐OVA (Figure S6A–C, Supporting Information). Importantly, sLNP‐OVA/Cel immunization led to a significant increase in the accumulation of Tregs in the lung by the end of the study (Figure 6D–F). These results indicate that our splenic DCs‐targeted tolerogenic mRNA‐LNPs vaccine could effectively mitigate the severity of allergic asthma by modulating the inflammatory microenvironment in the lungs.

**Figure 7:**
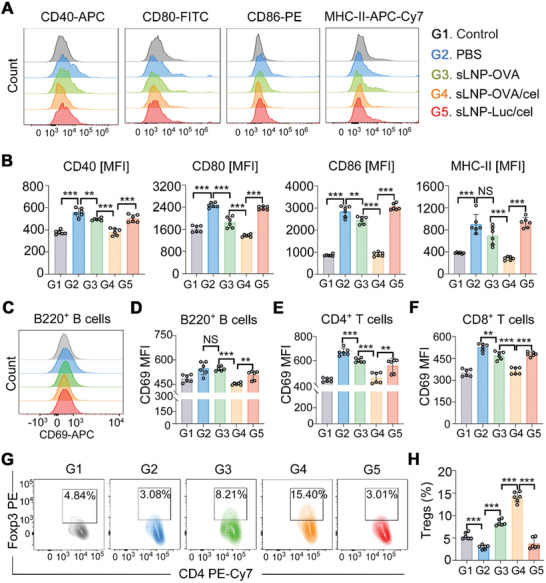
Systemic immunomodulatory effect of sLNP‐OVA/Cel in the spleen of asthma mice at the end of the study. A) Representative flow cytometry images showing CD40, CD80, CD86, and MHC‐II expression on splenic DCs from PBS and nanovaccine‐treated asthma mice. B) Quantitative analysis of (A). Error bars represent mean ± SEM (n = 6 biologically independent samples). C) Representative flow cytometry images demonstrating CD69 expression on splenic B cells from Control and nanovaccine‐treated asthma mice. D) Quantitative analysis of (C). Error bars represent mean ± SEM (n = 6 biologically independent samples). E,F) Quantitative analysis of CD69 expression on splenic CD4^+^ T cells (E) and CD8^+^ T cells (F), respectively. Error bars represent mean ± SEM (n = 6 biologically independent samples). G) Differentiation of Tregs in the spleen from PBS and nanovaccine‐treated asthma mice recorded by flow cytometry. H) Quantitative analysis of (G). Error bars represent mean ± SEM (n = 6 biologically independent samples). Statistical comparisons were made using one‐way ANOVA with Tukey's test. NS, no significance; **P < 0.01; ***P < 0.001.

Considering the immune cells’ reservoir role of the spleen, the activation of DCs and other immune cells such as T cells and B cells, and the number of splenic Tregs were accompanied by flow cytometry of splenic cell suspension at the end of the study. We observed that sLNP‐OVA immunization significantly suppressed the over‐activation of DCs (**Figure** **7**A,B), which correlated with the reduced lung inflammation score observed (Figure 5C). Moreover, sLNP‐OVA/Cel immunization further suppressed the over‐activation of DCs (Figure 7A,B), as well as B cells (Figure 7C,D), CD4^+^ T cells (Figure 7E), and CD8^+^ T cells (Figure 7F), compared to PBS or sLNP‐OVA treatment. Importantly, sLNP‐OVA/Cel immunization increased the number of splenic Tregs (Figure 7G,H), demonstrating its role in inducing a tolerogenic immune response in the spleen. Overall, the immunotherapeutic efficacy of the tolerogenic mRNA‐LNPs vaccine was accompanied by the induction of tolerogenic DCs and Tregs in the spleen, as well as an increase in the percentage of Tregs in the lung.

### 2.6 Immunotherapeutic Efficacy of Tolerogenic mRNA‐LNPs Vaccine Against Experimental Asthma after Sensitization

**Figure 8:**
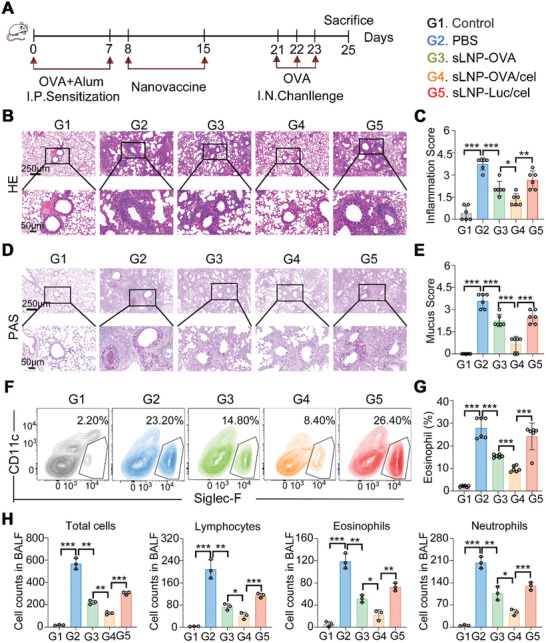
Prophylactic immunotherapy efficacy of sLNP‐OVA/Cel against experimental allergic asthma after sensitization. A) Schematic diagram illustrating the sensitization, immunization, and challenge protocol of the animals. B) Representative H&E staining images of lungs from PBS and nanovaccine‐treated asthma mice at the end of the study. Scale bars: 250 µm (top row) and 50 µm (bottom row). C) Quantitative analysis of peribronchial inflammation in (B). Error bars represent mean ± SEM (n = 6 biologically independent samples). D) Representative PAS staining images of lungs from Control and nanovaccine‐treated asthma mice at the end of the study. Scale bars: 250 µm (top row) and 50 µm (bottom row). E) Quantitative analysis of peribronchial mucus in (D). Error bars represent mean ± SEM (n = 6 biologically independent samples). F) Flow cytometric analysis of eosinophil infiltration in lungs from Control and nanovaccine‐treated asthma mice. G) Quantitative analysis of eosinophil infiltration in (F). Error bars represent mean ± SEM (n = 6 biologically independent samples). H) Total cell BALF and numbers of lymphocytes, eosinophils, and neutrophils. Error bars represent mean ± SEM (n = 3 biologically independent samples). Statistical comparisons were performed using one‐way ANOVA with Tukey's test. *P < 0.05; **P < 0.01; ***P < 0.001.

An important question is whether the generation of Tregs by sLNP‐OVA/Cel can prevent allergic asthma once allergic sensitization has occurred, even if prophylactic intervention before sensitization is observed. We investigated this by administering two intravenous doses of sLNP‐OVA, sLNP‐OVA/Cel, or sLNP‐Luc/Cel after two rounds of intraperitoneal sensitization (**Figure** **8**A). Subsequent intratracheal challenge induced asthma in untreated animals. Notably, both Cel‐loaded delivery platforms mitigated asthma development, with sLNP‐OVA/Cel demonstrating significantly greater efficacy than sLNP‐Luc/Cel. Suppression of asthma manifestations coincided with reduced lung inflammation (Figure 8B,C), mucus production (Figure 8D,E), and eosinophil infiltration (Figure 8F,G). Additionally, cell counts from BALF revealed significantly lower levels of total immune cells, lymphocytes, eosinophils, and neutrophils in the sLNP‐OVA/Cel group compared to PBS, sLNP‐OVA, or sLNP‐Luc/Cel treated groups (Figure 8H).

Furthermore, we evaluated this approach in a therapeutic model by administering mRNA vaccines after establishing experimental asthma (Figure S6D, Supporting Information). Lungs from PBS‐treated asthmatic mice exhibited pronounced inflammation (Figure S6E, Supporting Information). The inflammation score decreased in the sLNP‐OVA group compared to PBS‐treated groups (Figure S6F, Supporting Information). Importantly, the inflammation score of the sLNP‐OVA/Cel group was significantly lower than that of the sLNP‐OVA group (Figure S6F, Supporting Information), indicating the potent therapeutic effects of sLNP‐OVA/Cel against experimental allergic asthma. These findings highlight the potential of our splenic DCs‐targeted tolerogenic mRNA‐LNPs vaccine as a novel strategy for asthma treatment, warranting further research.

### 2.7 Tolerogenic mRNA‐LNPs Vaccine Exhibited Excellent Biocompatibility in Preliminary Safety Experiments

Lastly, we comprehensively evaluated the in vitro cytotoxicity and preliminary organ toxicity of sLNP‐OVA/Cel in vivo. Analysis of the cytotoxic effect of celastrol on BMDCs showed that even at high concentrations of up to 1 mg mL^−1^, celastrol did not induce significant cytotoxicity (Figure S7A, Supporting Information). Subsequently, BMDCs treated with PBS, sLNP‐OVA, sLNP‐OVA/Cel, or sLNP‐Cel for 4 h followed by LPS stimulation for 24 h showed no significant differences in cytotoxicity among the treatment groups (Figure S7B, Supporting Information). Histological examination of major organs at the end of the study on the generation of antigen‐specific Tregs in vivo revealed no signs of organ damage (Figure S8A, Supporting Information), indicating that intravenous administration of sLNP‐OVA/Cel did not result in overt organ toxicity. Additionally, there were no significant changes in the serum levels of alanine aminotransferase (ALT), aspartate aminotransferase (AST), total bilirubin (TBIL), blood urea nitrogen (BUN), albumin (ALB), and uric acid (UA) among mice treated with PBS, sLNP‐OVA, sLNP‐OVA/Cel, or sLNP‐Luc/Cel (Figure S8B, Supporting Information). These findings demonstrate the promising biocompatibility of our tolerogenic mRNA‐LNPs vaccine platform, supporting its further exploration in preclinical studies.

## 3 Discussion

mRNA vaccines have emerged as highly promising therapeutic tools for preventing a spectrum of diseases.^[^ ^1^, ^9^, ^10^ ^]^ However, their application in inducing immune tolerance presents significant challenges. These challenges primarily stem from the adjuvant properties of mRNA^[^ ^2^ ^]^ and the pro‐inflammatory nature of LNP carriers,^[^ ^24^ ^]^ which typically fail to elicit tolerogenic immune responses, particularly those mediated by tolerogenic DCs initiating Tregs. In this study, we deployed a novel tolerogenic mRNA vaccine strategy to design a splenic DCs‐selective LNP‐based tolerogenic mRNA vaccine delivery system that exploited the anti‐inflammatory potential of celastrol. The inclusion of celastrol was crucial for sLNPs to induce the differentiation of immature DCs into tolerogenic DCs, which serve as critical initiators of antigen‐specific tolerogenic immune responses. We further demonstrated that this celastrol‐based mRNA vaccine strategy effectively promoted the generation of splenic tolerogenic DCs and facilitated robust Tregs‐mediated tolerogenic immune responses. The potent immunoprophylactic, immunotherapeutic, and non‐toxic effects observed with our co‐delivery platform of nucleoside‐modified OVA‐mRNA and celastrol‐loaded sLNPs vaccine against experimental allergic asthma underscore the promising potential of this system for future clinical applications.

The rational formulation of nanoparticle vaccines has been demonstrated to finely regulate immunogenicity, either enhancing or suppressing antigen‐specific immune responses.^[^ ^14^, ^32^, ^41^, ^42^ ^]^ Targeting DCs to activate the immune system and induce antigen‐specific responses for treating tumors and infectious diseases has been extensively explored.^[^ ^33^, ^43^, ^44^ ^]^ In contrast to strategies focusing on immunoenhancement, herein, we investigate whether a celastrol‐loaded LNPs‐mRNA vaccine can induce tolerogenic DCs and promote the differentiation of Tregs to achieve antigen‐specific immunological tolerance, particularly in conditions of overactivated immune responses like asthma. Consistent with previous findings,^[^ ^45^ ^]^ we observed that celastrol‐loaded nanoparticles significantly inhibit DC maturation upon lipopolysaccharide stimulation. Incorporating celastrol into LNPs‐based mRNA vaccines enhances the differentiation of tolerogenic DCs, characterized by reduced expression of co‐stimulatory proteins and increased production of immunosuppressive cytokines, akin to the effects of glucocorticoids and other immunomodulators.^[^ ^19^, ^46^, ^47^ ^]^ It has been reported that celastrol can inhibit pro‐inflammatory cytokine production by inhibiting the TLR4‐MyD88‐NF‐κB pathway^[^ ^48^ ^]^ or ROS‐NF‐κB inflammasome axis,^[^ ^49^ ^]^ while promoting the production of anti‐inflammatory cytokines of IL‐10.^[^ ^50^ ^]^ Whether celastrol plays a unique role in tolerogenic DC differentiation or if other anti‐inflammatory agents could potentially yield similar or superior effects, warrants further investigation. As for tolerogenic mRNA delivery vectors, the safety and efficacy of ionizable lipid of SM102‐based LNPs in protecting and delivering mRNA have been extensively demonstrated, especially in human beings. SM102‐based mRNA‐lipid nanoparticles vaccines have been approved for combating SARS‐CoV‐2, which contribute to the development of LNPs‐based tolerogenic mRNA vaccine. The mechanisms of tolerogenic mRNA vaccines and antiviral mRNA vaccines are different, and the research content of antiviral vaccines is not entirely applicable to tolerant vaccines. Therefore, there remains a lot of work for developing LNPs based tolerogenic mRNA. While further research is necessary, our proof‐of‐concept study demonstrates that formulation adjustments can counteract the pro‐inflammatory adjuvant activity of stearic acid‐doped LNPs, thereby facilitating the differentiation of immature DCs into tolerogenic DCs. This approach may establish a novel paradigm for the development of LNPs‐based tolerogenic mRNA vaccines.

Tolerogenic mRNA vaccines have recently emerged as promising candidates for therapeutic applications, demonstrating both safety and efficacy in various preclinical models.^[^ ^3^, ^15^ ^]^ However, one concern exists regarding the large amount of mRNA needed to induce potent therapeutic effects, such as the mRNA dosage used by Andre E Nel et al. was 1.25 mg kg^−1^ (≈25 µg mouse^−1^)^[^ ^15^ ^]^ and the mRNA dosage used by Ugur Sahin et al. was as high as 40 µg mouse^−1^.^[^ ^3^ ^]^ To address this challenge, splenic DC‐targeted mRNA vaccines were developed to reduce mRNA dosage, potentially advancing vaccine efficacy and safety. In our experiments investigating immunoprophylaxis against experimental asthma, the splenic DC‐targeted tolerogenic mRNA vaccines effectively suppressed asthma progression using a reduced mRNA dosage of 0.5 mg kg^−1^ (≈10 µg mouse^−1^). DCs, pivotal in antigen presentation, play a critical role in stimulating antigen‐specific Tregs that modulate immune responses.^[^ ^3^, ^39^ ^]^ Our findings demonstrate that sLNPs‐OVA/Cel induced significantly higher numbers of antigen‐specific CD4^+^ Tregs in the spleen compared to sLNPs‐OVA. This efficient mRNA vaccine delivery to splenic APCs likely underlies the observed potent anti‐asthma efficacy, primarily attributed to the induction of splenic tolerogenic DCs and subsequent antigen‐specific Tregs. Preliminary safety assessments revealed no significant cytotoxicity in vitro or organ toxicity in vivo for the celastrol‐assisted splenic DCs‐targeted mRNA vaccine. These results collectively highlight the promising feasibility of splenic DCs‐targeted tolerogenic mRNA vaccines, combining potent tolerogenic immunotherapy efficacy with excellent biocompatibility. Further research is essential to refine and optimize this vaccine strategy for potential clinical translation.

While our study demonstrates the prophylactic and therapeutic efficacy of sLNPs‐OVA/Cel in an experimental allergic asthma model, several limitations should be acknowledged. The use of the OVA allergen, while standard in asthma research,^[^ ^51^, ^52^, ^53^, ^54^, ^55^ ^]^ does not fully reflect the complexity of allergens encountered in real‐world asthma. Future efforts should focus on designing allergens that better mimic clinical conditions to ensure robust and effective induction of tolerogenic immune responses. Additionally, the mechanisms underlying spleen‐selective mRNA translation by stearic acid‐doped LNPs require further elucidation. Our previous work suggests that reduced accumulation of sLNPs in the liver may contribute to exclusive extrahepatic protein expression in the spleen.^[^ ^34^ ^]^ However, factors such as lysosomal escape^[^ ^56^ ^]^ and intracellular mRNA transport^[^ ^57^ ^]^ likely play crucial roles in mediating this selective translation. Future studies should aim to unravel these mechanisms to optimize the design of spleen‐targeted mRNA vaccines. Furthermore, while our study provides a promising proof‐of‐concept, validation in additional animal models, including non‐human primates, is essential to advance this novel tolerogenic vaccine toward clinical application in asthma prevention and treatment. The celastrol‐loaded sLNP formulation developed here, capable of encoding various allergenic or autoimmune antigens, holds potential as a universal platform for tolerogenic mRNA vaccine delivery. Taken together, our findings underscore the significance of developing effective immunotherapies for allergic diseases amidst a global rise in prevalence and addressing these research gaps and optimizing our approach could pave the way for impactful clinical interventions in the near future.

## 4 Conclusions

In conclusion, we developed a spleen‐targeted tolerogenic mRNA‐LNP vaccine platform that incorporates nucleoside‐modified mRNA and the anti‐inflammatory agent celastrol. This platform effectively induces tolerogenic DCs and antigen‐specific Tregs within the spleen, thereby mitigating allergic responses to the model antigen OVA. Our immunotherapy approach not only promoted the generation of antigen‐specific Tregs in the spleen but also facilitated their migration to the lungs, where they suppressed Th2 immunity. Given the systemic immunoregulatory effects of the spleen and the platform's versatility, it holds promise for therapeutic interventions in diverse immune disorders, including autoimmunity, organ transplantation, chronic infections, and allergic diseases.

## 5 Experimental Section

### Materials

SM102, DSPC, DMG‐PEG and cholesterol were purchased from Shanghai A.V.T. Pharmaceutical Co., Ltd (Shanghai, China). Citrate buffer and phosphate buffer (PBS) were purchased from Solorbio (Beijing, China). Stearic acid, bovine serum albumin (BSA), and ovalbumin (OVA) were supplied by Sigma–Aldrich (USA). The T7 High Yield RNA Transcription Kit, N1‐Me‐Pseudo UTP, Cap 1 Capping System, and E.coli Poly(A) Polymerase were acquired from Novoprotein (China). D‐Luciferin (sodium salt) was purchased from Biovision Biotechnology (USA).

The antibodies used for flow cytometry were listed in Extended Data Table S1 (Supporting Information), and the sequences of all primers utilized for qRT‐PCR were shown in Extended Data Table S2 (Supporting Information). ELISA kits for Mouse IL‐10 (EK0417), Mouse TGF‐β (EK0515), Mouse IL‐4 (EK0405), Mouse IL‐5 (EK0408), and Mouse IL‐13 (EK0425) were procured from BOSTER Biological Technology Co., Ltd. (China). Mouse anti‐IgE antibodies were sourced from Invitrogen (USA). Antibodies specific for Ikkβ (8943S), p‐Ikkβ (2697S), p65 (3033S), and p‐p65 (8242S) were purchased from Cell Signaling Technology. HRP‐conjugated anti‐rabbit antibody (AS014) was obtained from ABclonal, and antibodies against ACTB (GB11001‐100) were acquired from Servicebio (China).

### Preparation of Nucleoside‐Modified mRNA

Nucleoside‐modified OVA‐mRNA and Luc‐mRNA were synthesized using T7 RNA polymerase (Novoprotein) with N1‐Me‐Pseudo UTP substituted for UTP, following the manufacturer's instructions. All mRNAs were stored frozen at −80 °C.

### Preparation and Characterization of the Tolerogenic mRNA Vaccine

The splenic DCs‐targeted mRNA‐loaded LNPs were prepared as previously described.^[^ ^34^ ^]^ Briefly, an ethanolic lipid solution containing SM‐102, DSPC, cholesterol, DMG‐PEG, and stearic acid was combined with an aqueous solution of mRNA in citrate buffer (pH 4.5) by rapid pipetting. LNPs loaded with celastrol were prepared similarly, with celastrol (1% of the lipid mass) added to the ethanolic lipid solution. The final sLNPs‐OVA and sLNPs‐OVA/Cel formulations were obtained by dialysis against 1× PBS.

The mRNA loading efficiency of sLNPs‐OVA and sLNPs‐OVA/Cel was initially assessed using denaturing formaldehyde agarose gel electrophoresis. Quantitative mRNA encapsulation efficiency was determined using the Quant‐iT RiboGreen RNA assay Kit. Particle size and zeta potential of sLNPs‐OVA and sLNPs‐OVA/Cel were measured using a Zetasizer Nano ZS90 (Malvern Instruments, Malvern, United Kingdom). The morphology of the nanoparticles was examined by transmission electron microscopy (Hitachi, Tokyo, Japan).

### Splenic DCs Selectivity and Suppression of Splenic Immune Cell Activation by Tolerogenic mRNA Vaccine In Vivo

The distribution of Cy5‐labeled sLNPs‐Luc/Cel in the spleen was analyzed ex vivo 6 h after intravenous injection. The expression of luciferase and the fluorescent signal density of LNPs‐based mRNA vaccine delivery systems in the spleen were quantified using PerkinElmer equipment. Single‐cell suspensions were prepared from a portion of the spleen and analyzed by flow cytometry. Splenic cell subsets were labeled with antibodies against CD45, CD3, B220, CD11b, and CD11c, with DAPI used for viability staining to exclude dead cells. The proportions of Cy5^+^/CD11c^+^ DCs, Cy5^+^/CD11b^+^ macrophages, Cy5^+^/CD3^+^ T cells, and Cy5^+^/B220^+^ B cells were determined. Additionally, frozen spleen sections from another portion of the spleen were acquired and stained with DAPI and CD11c to investigate the intrasplenic spatial distribution of the tolerogenic mRNA‐LNPs vaccine.

To investigate the induction of tolerogenic splenic DCs in vivo, BALB/c mice were intravenously injected with PBS, sLNPs‐OVA, sLNPs‐OVA/Cel (at mRNA dosages of 0.5 mg kg^−1^), or sLNPs‐Cel. Spleens were excised 24 h post‐injection, and splenic cell subsets were labeled with antibodies against CD11c, CD40, CD80, CD86, MHC‐II, CD4, CD8, B220, and CD69. Flow cytometry was used to determine the proportions of CD40^+^, CD80^+^, CD86^+^, or MHC‐II^+^ cells within the CD11c^+^ DC population, as well as CD69 expression in CD4^+^ T cells, CD8^+^ T cells, and B220^+^ B cells. Data analysis was performed using FlowJo software (V10).

### Differentiation of Tolerogenic BMDCs from Immature BMDCs In Vitro

BMDCs were prepared as previously described.^[^ ^32^ ^]^ Briefly, bone marrow cells were obtained from 6 to 8‐week‐old female mice on Day 0 and cultured in RPMI 1640 supplemented with 10% heat‐inactivated FBS, 100 U mL^−1^ penicillin, 100 µg mL^−1^ streptomycin, 55 µM β‐mercaptoethanol, and 20 ng mL^−1^ GM‐CSF. After three days, an equal volume of complete medium with supplements was added, and on Day 7, suspended cells were used for experiments.

To assess the tolerogenic characteristics of BMDCs treated with sLNP‐OVA/Cel followed by LPS stimulation, the following procedures were performed: After the incubation period, the cells were harvested by scraping and then treated with FACS buffer (PBS containing 2% FBS). They were subsequently stained with antibodies against CD11c, CD40, CD80, CD86, IL‐10, and TGF‐β at 4 °C for 30 min. After staining, the cells were washed twice with FACS buffer and resuspended in FACS buffer using a BD flow cytometer, and the data were assessed using FlowJo V10.

Gene expression analysis in BMDCs treated with sLNPs‐OVA/Cel (or sLNPs‐OVA) was conducted using RNA sequencing. RNA samples were extracted and processed for sequencing at OE Biotech Co., Ltd. (Shanghai, China) using a BGI sequencer. The DESeq2 software was used for data analysis to identify differential gene expression patterns. Additionally, a portion of the extracted RNA samples was used to investigate the transcription levels of pro‐inflammatory and anti‐inflammatory cytokines.

Whole‐cell lysates from BMDCs treated with sLNP‐OVA/Cel followed by stimulation with LPS were analyzed for the activation of the NF‐κB signaling pathway. Protein samples were subjected to Western blotting by incubating with a 1:10 000 dilution of monoclonal antibodies against p65, phosphorylated p65 (pp65), IKKβ, or phosphorylated IKKβ (pIKKβ) overnight at 4 °C. Subsequently, donkey anti‐mouse or anti‐rabbit horseradish peroxidase‐conjugated secondary antibodies (1:5000 dilution) were used to detect the primary antibody signals. The blots were visualized using the Amersham ECL Western Blotting Detection Reagent and an Amersham Imager 600 system (both from GE Healthcare).

### Functional Treg Induction In Vitro and In Vivo

To assess the ability of whether celastrol addition in the mRNA‐LNPs vaccine platform could facilitate the differentiation of Tregs from naïve CD4^+^ T cells in vitro, splenocyte suspensions from 6 to 8‐week‐old female BALB/c mice were used to isolate CD4^+^ T cells via magnetic beads. BMDCs from 6 to 8‐week‐old female BALB/c mice were treated with different formulations for 4 h followed by stimulation with LPS for 24 h. These cells were then co‐cultured with splenic CD4^+^ T cells for 72 h. Non‐adherent cells were harvested and stained with anti‐mouse CD4, anti‐mouse Foxp3, IL‐10, and TGF‐β antibodies. Flow cytometry analysis was performed to quantify the differentiation of CD4^+^ T cells into Tregs induced by BMDCs treated with tolerogenic nano vaccines. Additionally, qRT‐PCR was employed to assess the mRNA expression levels of Foxp3, IL‐10, TGF‐β, and IFN‐γ in the non‐adherent Tregs.

The functional suppression assays of Tregs generated from sLNP‐OVA/Cel‐treated BMDCs were further explored. Specifically, splenic cells from normal mice were stained with CFSE and co‐incubated with Tregs generated from sLNP‐OVA/Cel‐treated BMDCs for 3 days. CFSE staining was performed to rule out Treg generated from sLNP‐OVA/Cel‐treated BMDCs. To assess the differentiation of naive CD4^+^ T cells into Th2 cell clusters, cells were restimulated with Cell Activation Cocktail (PMA/Ionomycin with Brefeldin A) for 4 h. After washing twice with PBS, FVD staining was performed at a 1:1000 dilution to exclude dead cells. Surface antibody staining was conducted at a 1:100 dilution against CD4, followed by intracellular IL‐4 staining after fixation and permeabilization.

To investigate the induction of Tregs, antigen‐specific Tregs, and memory T cells in vivo, BALB/c mice received intravenous injections of PBS, sLNPs‐OVA, sLNPs‐OVA/Cel, or sLNPs‐Luc/Cel (mRNA dosages of 0.5 mg kg^−1^) on days 0 and 7. On day 14, spleen and lung tissues were collected. Erythrocytes were lysed using red blood cell lysis buffer, and splenic or lung cell subsets were labeled with antibodies against B220, CD4, and Foxp3, as well as the H‐2IAd/AAHAEINEA tetramer (OVA329‐337 tetramer). Additionally, splenic cell subsets were stained with CD4, CD8, CD44, and CD62L antibodies to analyze effector memory T cells and central memory T cells. Flow cytometry was performed to assess the Treg population (B220‐CD4^+^Foxp3^+^) and antigen‐specific Treg population (H‐2IAd/AAHAEINEA^+^/B220‐CD4^+^Foxp3^+^). Data analysis was performed using FlowJo software (V10).

### Experimental Allergic Asthma Mouse Model

BALB/c mice were purchased from Vital River Laboratory Animal Technology Co., Ltd (Beijing, China) and housed in a specific pathogen‐free environment at the Experimental Animal Facility, Academy of Medical Science, Zhengzhou University. All animal procedures were approved by the First Affiliated Hospital of Zhengzhou University Animal Care and Use Committee (Protocol No. 2021‐KY‐0634‐001). To induce experimental allergic asthma, BALB/c mice were sensitized intraperitoneally with 50 µg of OVA with aluminum adjuvant once a week for two weeks. Two weeks after the final sensitization, mice were challenged intranasally with 20 µg of OVA daily for three consecutive days.

### Immunization

For the prophylactic studies in experimental asthma, BALB/c mice received intravenous injections of PBS, sLNPs‐OVA, sLNPs‐Luc/Cel, or sLNPs‐OVA/Cel (mRNA dosages of 0.5 mg kg^−1^) on days 0 and 7. On day 14, mice were intraperitoneally sensitized with ovalbumin once a week for two weeks. Subsequently, mice were intratracheally challenged with OVA to induce asthma models for three consecutive days starting on day 35. Animals were euthanized 2 days after the final intratracheal challenge, and lung tissues were harvested post‐mortem. BALF was collected by lavaging the lungs with 1 mL of PBS supplemented with 2% newborn calf serum twice. BALF IgE levels and cytokine profiles were quantified using ELISA following the manufacturer's protocols.

In the therapeutic vaccination model following sensitization, 6‐8‐week‐old BALB/c mice received two doses of sLNP‐OVA, sLNP‐OVA/Cel, or sLNP‐Luc/Cel via intravenous injection once a week, commencing one day after two rounds of intraperitoneal sensitization with ovalbumin. Six days after the final immunization, mice were intratracheally challenged with OVA to induce asthma models for three consecutive days. Animals were euthanized 2 days after the last intratracheal challenge for subsequent analysis.

### Histology and Immune Microenvironment of the Lungs

The lung tissues were harvested from each mouse at the end of the study. Then, half of each lung was embedded in paraffin and serially sectioned frontally at 5 µm thickness. Inflammatory cell infiltration was assessed using hematoxylin and eosin (H&E) staining, while mucus secretion was visualized with periodic acid‐Schiff (PAS) staining and photographed using a Slide Scan System SQS (TEKSQRAY). Additionally, infiltration of inflammatory monocytes was evaluated using anti‐CD11b primary antibody followed by fluorescence‐labeled secondary antibody staining. Similarly, the presence of Tregs was examined with anti‐Foxp3 primary antibody and fluorescence‐labeled secondary antibody staining. Imaging was performed using a Perkin Elmer Vectra machine. Furthermore, single‐cell suspensions of lung tissues were stained with CD45, CD11b, CD4, and Foxp3 antibodies and analyzed by flow cytometry.

### In Vitro and In Vivo Toxicity of the Tolerogenic mRNA Vaccine

BMDCs were seeded at a density of 1 × 10^4^ cells well^−1^ and treated with varying concentrations of celastrol for 24 h at 37 °C and 5% CO2. Cell viability was assessed using a CCK8 kit. The cytotoxicity of sLNP‐OVA/Cel on BMDCs was evaluated similarly to the differentiation of tolerogenic DCs, also employing the CCK8 kit.

Organs, including the liver, spleen, lungs, kidneys and heart, were excised from BALB/c mice at the conclusion of OVA‐specific Treg immune response experiments. These organs were immediately fixed in PBS containing 4% paraformaldehyde for three days, embedded in paraffin, and sectioned transversely at 5 µm thickness. Inflammatory tissue reactions were examined using H&E staining. Blood samples were collected for measurement of plasma alanine aminotransferase (ALT), aspartate aminotransferase (AST), total bilirubin (TBIL), blood urea nitrogen (BUN), albumin (ALB), and uric acid (UA) levels.

### Statistical Analysis

Quantitative data were presented as mean ± SEM unless otherwise specified. Statistical analyses of data from two or multiple groups were performed using two‐sided Student's t‐test or nonparametric one‐way ANOVA with Tukey's test, respectively, using GraphPad Prism 8.0. Statistical significance was indicated as follows: *P < 0.05, **P < 0.01, ***P < 0.001, and NS indicating no significant differences.

## Conflict of Interest

The authors declare no conflict of interest.

## Supporting information



Supporting Information

## Data Availability

The data that support the findings of this study are available from the corresponding author upon reasonable request.
