# Supplementary material for: A Spleen‐Targeted Tolerogenic mRNA‐LNPs Vaccine for the Treatment of Experimental Asthma
Source: Adv Sci (Weinh). 2025 Feb 8;12(13):2412543. doi: 10.1002/advs.202412543 (PMC11967843; doi:10.1002/advs.202412543)
Supplement: Supplementary file 1 — Supporting Information [file ADVS-12-2412543-s001.docx]

Supporting Information

**A Spleen-Targeted Tolerogenic mRNA-LNPs Vaccine for the Treatment of Experimental Asthma**

*Fazhan Wang*, Jia Lou, Xiaohan Lou, Fang Wu, Xiaoke Gao, Xiaohan Yao, Jiajia Wan, Xixi Duan, Wenjing Deng, Lixia Ma, Lijing Zhang, Guangjie He, Ming Wang, Chen Ni, Ningjing Lei*, Zhihai Qin**

## Tables

**Table 1. List of antibodies for flow cytometry in this study.**

| Target | Colour | Clone | Manufacturer | Lot No. |
| --- | --- | --- | --- | --- |
| CD45 | Alexa Fluor® 700 | I3/2.3 | Biolegend | 147716 |
| CD45R/B220 | PerCP/Cyanine5.5 | RA-36B2 | Biolegend | 103236 |
| CD3 | PE/Cyanine 7 | 17A2 | Biolegend | 100220 |
| CD11b | PE | M1/70 | Biolegend | 101208 |
| CD11b | FITC | M1/70 | Biolegend | 101206 |
| CD11c | FITC | N418 | Biolegend | 117306 |
| CD11c | PerCP/Cyanine5.5 | N428 | Biolegend | 117327 |
| CD40 | FITC | HM40-3 | Biolegend | 102905 |
| CD80 | APC | 16-10A1 | Biolegend | 104714 |
| CD86 | PE | A17199A | Biolegend | 159204 |
| I-Ab | APC/Cyanine7 | AF6-120.1 | Biolegend | 116426 |
| I-A/I-E | APC/Cyanine7 | M5/114.15.2 | Biolegend | 107627 |
| CD4 | PE Cyanine 7 | GK1.5 | Biolegend | 100422 |
| CD4 | PerCP/Cyanine5.5 | RM4-5 | Biolegend | 100539 |
| CD8a | PE | 53-6.7 | Biolegend | 100707 |
| CD8a | FITC | 53-6.7 | Biolegend | 100706 |
| CD69 | APC | H1.2F3 | Biolegend | 104514 |
| IL-10 | FITC | JES5-16E3 | Biolegend | 505006 |
| IL-4 | PE | 11B11 | Biolegend | 504103 |
| LAP (TGF-β1) | PE-Cyanine 7 | TW7-16B4 | invitrogen | 25-9821-82 |
| Foxp3 | Alexa Fluor® 488 | [MF-14](https://www.biolegend.com/en-us/search-results?Clone=MF-14) | Biolegend | 126406 |
| Foxp3 | PE | MF-14 | Biolegend | 126403 |
| CD45R/B220 | Brilliant Violet 510™ | RA3-6B2 | Biolegend | 103248 |
| CD170 (Siglec-F) | PE | S17007L | Biolegend | 155506 |
| CD44 | PE | IM7 | Biolegend | 103008 |
| CD62L | APC | MEL-14 | Biolegend | 104412 |
| ProT2 MHC-II Tetramers | APC |  | Proimmune | TT3223A |

**Table 2. Primers used for qRT-PCR.**

| Gene | Primer sequence (5’-3’) |
| --- | --- |
| IL10 | 5’-CCCATTCCTCGTCACGATCTC-3’  5’-TCAGACTGGTTTGGGATAGGTTT-3’ |
| TGF-β | 5’- AAGTTGGCATGGTAGCCCTT-3’  5’- GGAGAGCCCTGGATACCAAC-3’ |
| IL6 | 5’- TAGTCCTTCCTACCCCAATTTCC-3’  5’- TTGGTCCTTAGCCACTCCTTC-3’ |
| IL1β | 5’- TTCAGGCAGGCAGTATCACTC-3’  5’- GAAGGTCCACGGGAAAGACAC-3’ |
| Foxp3 | 5’- CCTGCCTTGGTACATTCGTG-3’  5’- TGTTGTGGGTGAGTGCTTTG-3’ |
| IFN-γ | 5’- ATGAACGCTACACACTGCATC-3’  5’- ACCTGTGGGTTGTTGACCTCA-3’ |
| GAPDH | 5’-TGTGTCCGTCGTGGATCTGA-3’  5’-CCTGCTTCACCACCTTCTTGAT-3’ |

## Figures


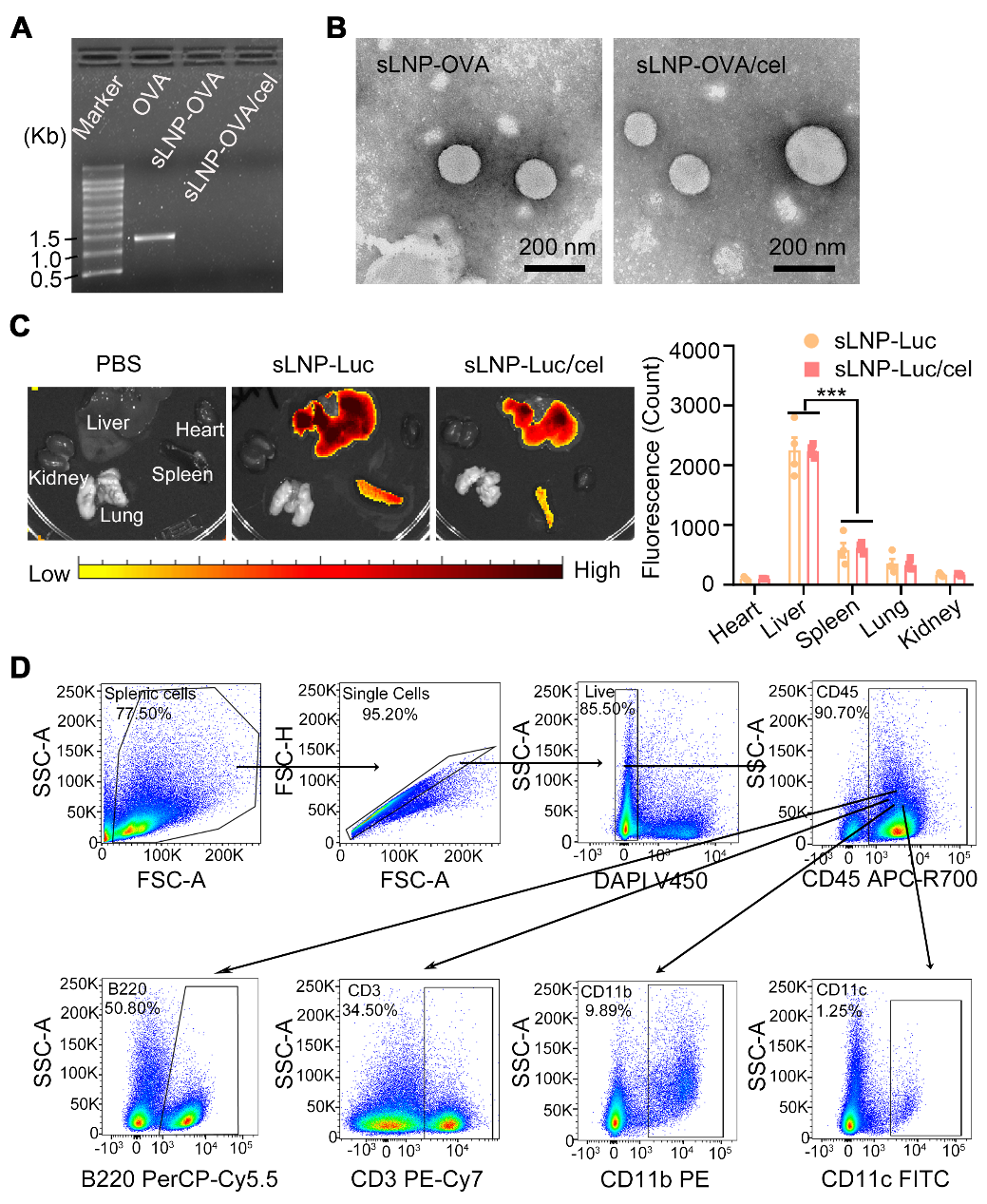


**Figure S1. Characterization of the splenic DCs-targeted LNPs-based tolerogenic mRNA vaccine delivery systems.** A, Loading capacity of nucleoside-modified mRNA by the sLNP co-loaded with or without celastrol determined via denatured agarose gel electrophoresis. B, Morphology of sLNP-based mRNA vaccine delivery systems loaded without (left) or with (right) celastrol observed using transmission electron microscopy. C, Biodistribution of sLNP co-loaded with or without celastrol in the major organs of BALB/c mice 6 h after intravenous injection. D, Gating strategy for cellular uptake of sLNP-based mRNA vaccine delivery systems by splenic cell types. Comparisons were made using two-sided Student's t-test. ***P < 0.001.


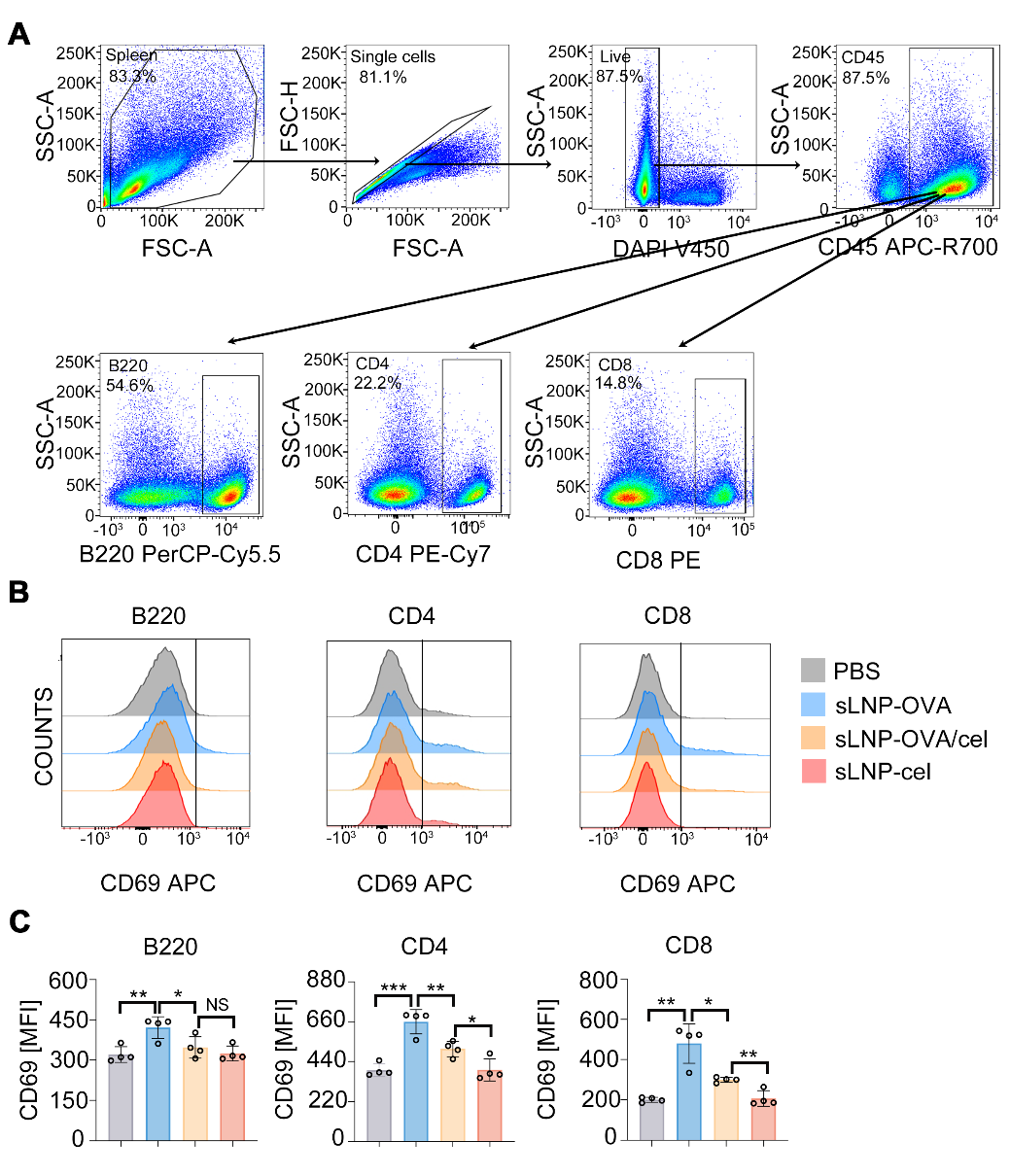


**Figure S2. Activation of splenic B cells and T cells from mice treated with or without celastrol-loaded vaccine delivery systems 24 h after intravenous injection.** A, Gating strategy for activation of different splenic cell types. B, Representative flow cytometry images of CD69 expression in B cells, CD4 T cells, and CD8 T cells. C, Quantitative analysis of (B). Error bars represent mean ± SEM (n = 4 biologically independent samples). Comparisons were made using one-way ANOVA with Tukey's test. NS, no significance; *P < 0.05; **P < 0.01; ***P < 0.001.


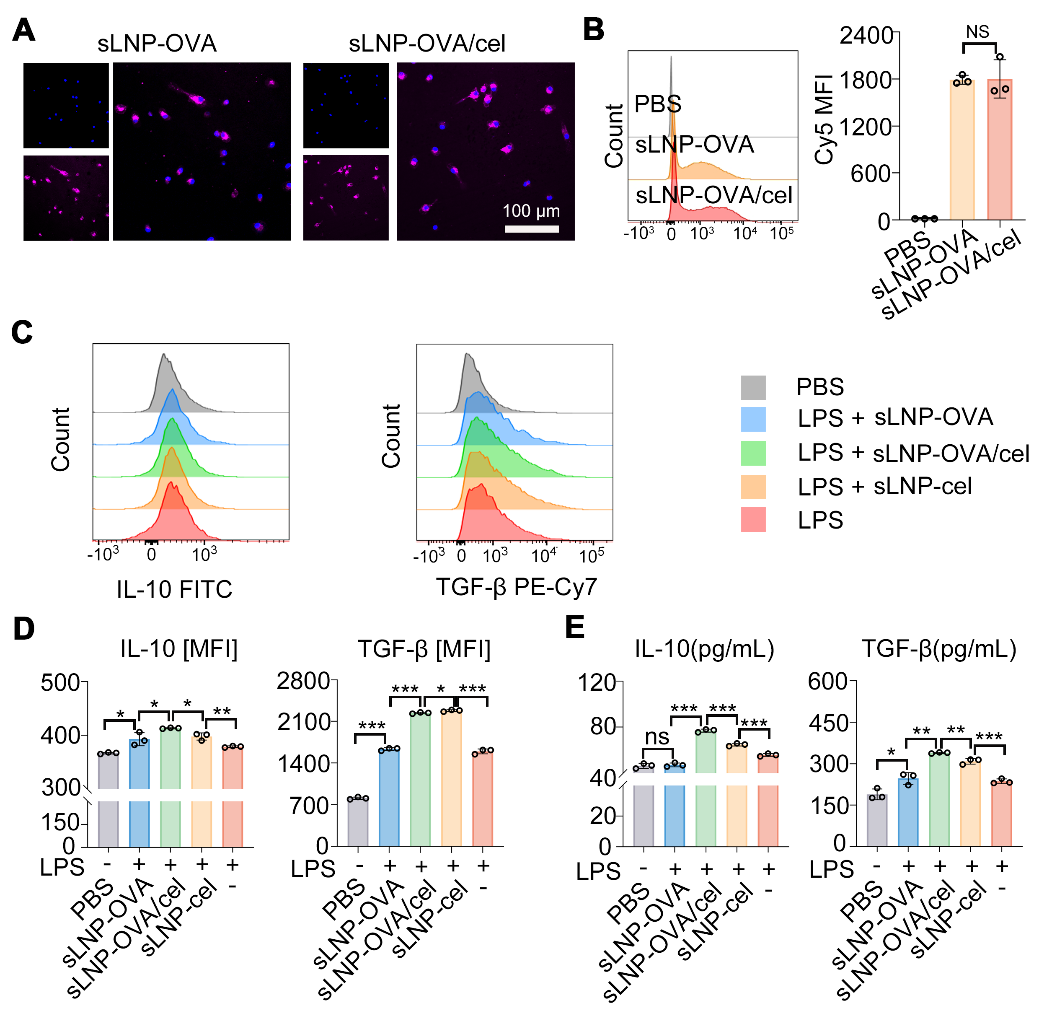


**Figure S3. Inclusion of celastrol into nucleoside-modiﬁed mRNA-LNPs delivery systems facilitated the secretion of immunosuppressive cytokines.** A, Representative confocal images showing uptake of sLNPs-based tolerogenic mRNA vaccine by BMDCs. B, Quantification of cellular uptake of the tolerogenic mRNA vaccine by flow cytometry. Error bars represent mean ± SEM (n = 3 biologically independent samples). C, Representative flow cytometry images of IL-10 and TGF-β expression on BMDCs. D, Quantitative analysis of the mean fluorescence intensity (MFI) of IL-10 and TGF-β on BMDCs. Error bars represent mean ± SEM (n = 3 biologically independent samples). E, Levels of immunosuppressive cytokines IL-10 and TGF-β secreted from BMDCs. Error bars represent mean ± SEM (n = 3 biologically independent samples). Data in b were analyzed using two-sided Student's t-test. Comparisons for d and e were made using one-way ANOVA with Tukey's test. NS, no significance; *P < 0.05; **P < 0.01; ***P < 0.001.


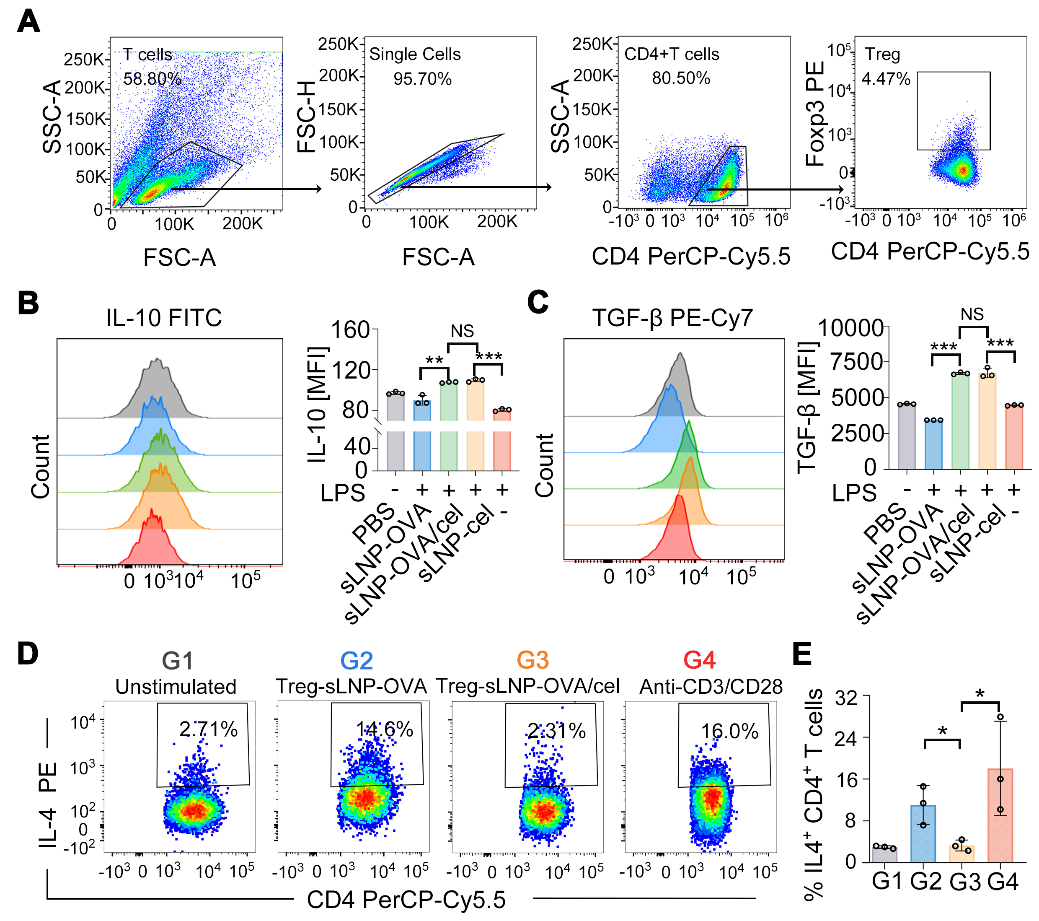


**Figure S4. Secretion of immunosuppressive cytokines by induced Tregs in vitro and suppression functional assays of Tregs generated from sLNP-OVA/Cel-treated BMDCs.** A, Gating strategy for differentiation of naïve CD4+ T cells into CD4+ Tregs in vitro. B, C, Representative flow cytometry images and quantitative analysis of IL-10 (B) and TGF-β (C) expression in induced CD4+ Tregs. Error bars represent mean ± SEM (n = 3 biologically independent samples). D, Suppression function of Tregs on the differentiation of naive CD4+ T cells into T helper 2 (IL-4-producing T cell, Th2) cells recorded by flow cytometry. E, Quantitative analysis of the proportion of IL-4-producing T cells among CD4+ T cells. Error bars represent mean ± SEM (n = 3 biologically independent samples). Data in B–C and E–E were analyzed using one-way ANOVA with Tukey's test and a two-sided Student's t-test, respectively. NS, no significance; *P < 0.05; **P < 0.01; ***P < 0.001.


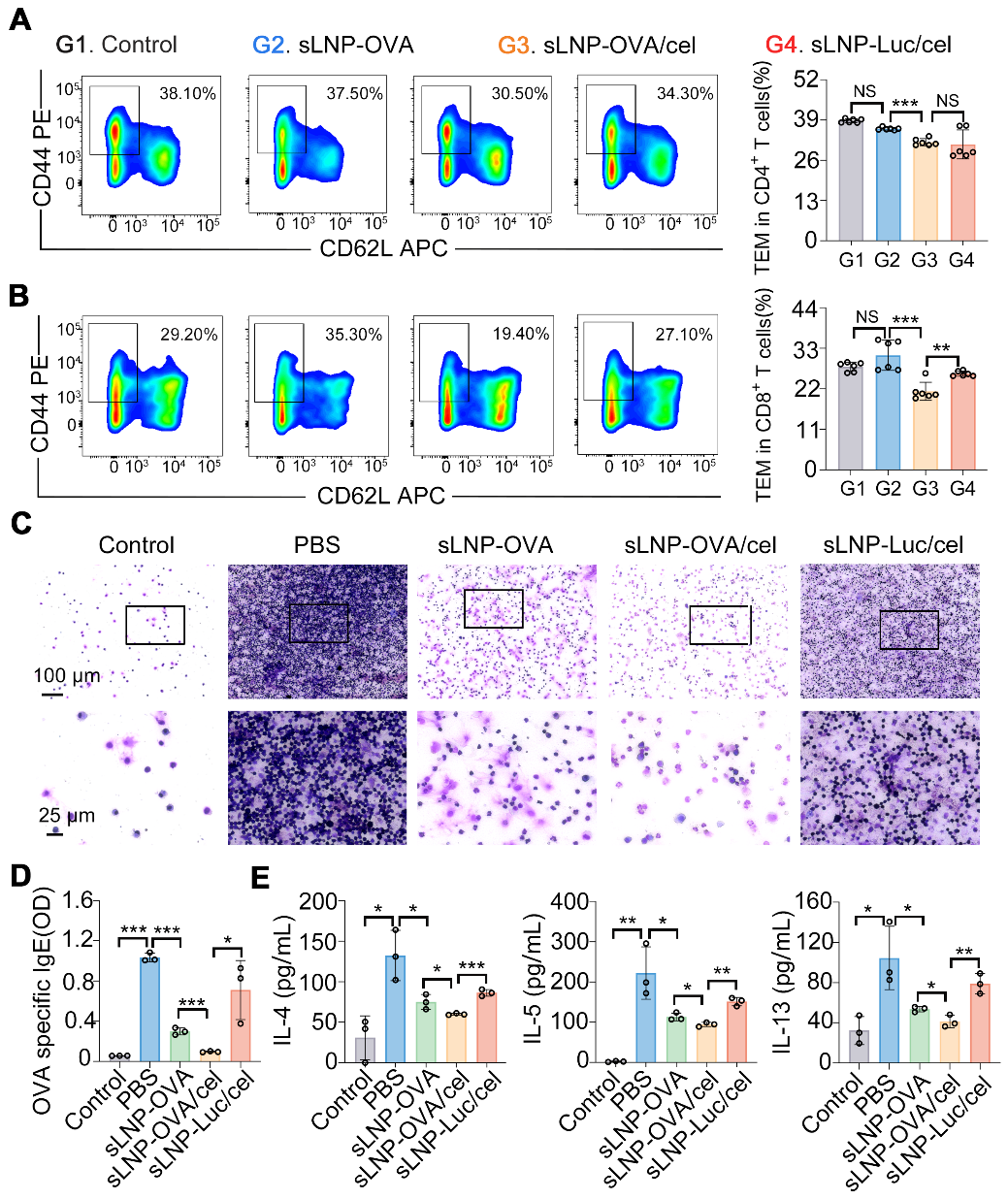


**Figure S5. Effector memory T cells in spleen one week after two immunizations and analysis of BALF at the end of the prophylactic study.** A, Representative flow cytometry images, and quantitative analysis of effector memory T cells (TEM) in CD4+ T cells. Error bars represent mean ± SEM (n = 6 biologically independent samples). B, Representative flow cytometry images and quantitative analysis of TEM in CD8+ T cells. Error bars represent mean ± SEM (n = 6 biologically independent samples). C, Representative images of Rui's Giemsa staining of BALF at the end of the prophylactic experimental asthma. D, E, Levels of OVA-specific IgE in BALF (D) and Th2-associated cytokines IL-4, IL-5, and IL-13 in BALF (E) at the end of the prophylactic experiment against asthma. Error bars represent mean ± SEM (n = 3 biologically independent samples). Comparisons were made using one-way ANOVA with Tukey's test. NS, no significance; *P < 0.05; **P < 0.01; ***P < 0.001.


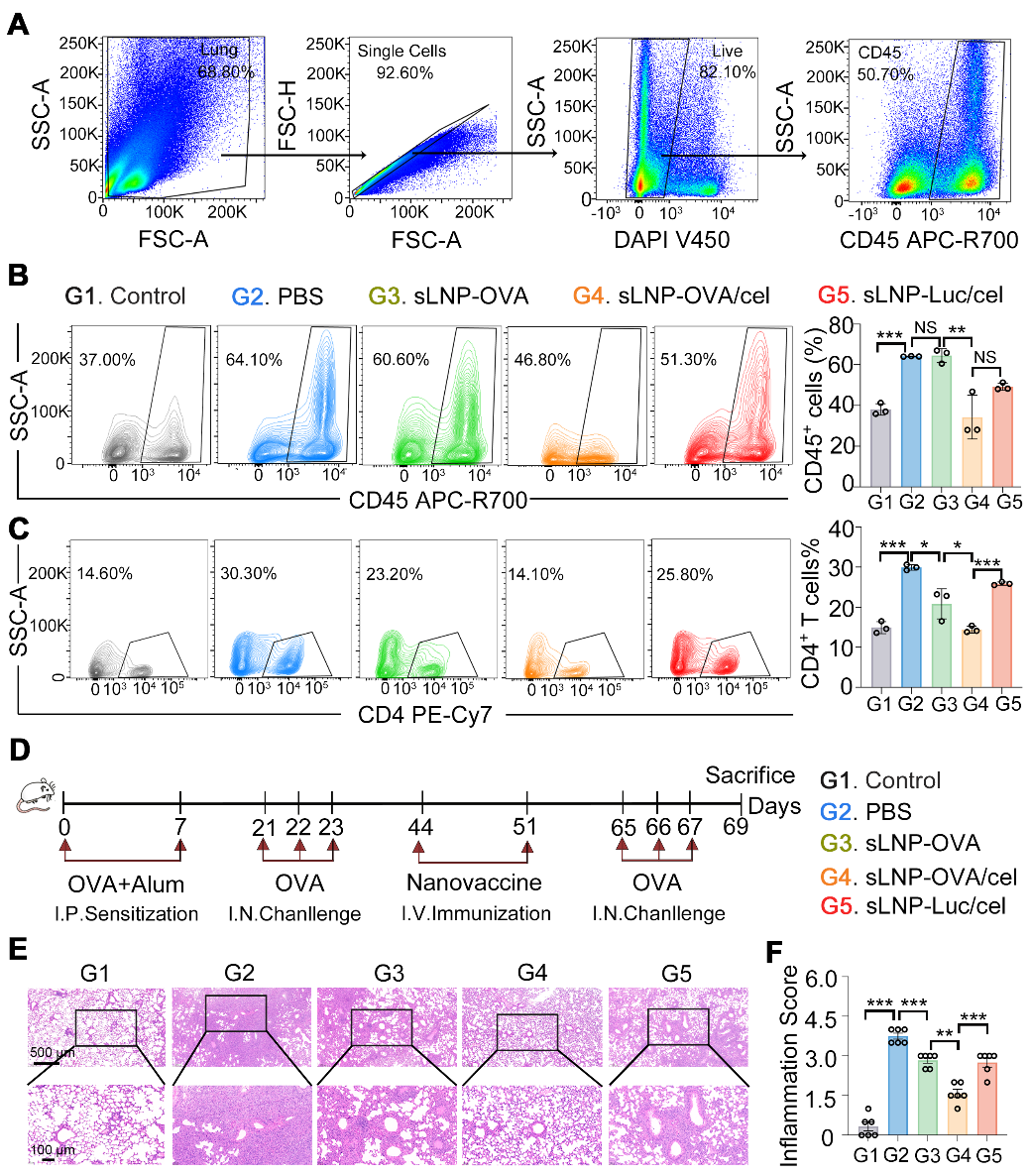


**Figure S6. The infiltration of CD45+ immune cells and CD4+ T cells in the lung at the end of the prophylactic experimental asthma and therapeutic effects of sLNP-OVA/Cel against experimental allergic asthma.** A, Gating strategy for CD45+ immune cells in the lung. B, Quantitative analysis of CD45+ cells. Error bars represent mean ± SEM (n = 3 biologically independent samples). C, Quantitative analysis of CD4+ T cells. Error bars represent mean ± SEM (n = 3 biologically independent samples). D, Schematic diagram illustrating the intraperitoneal sensitization, intratracheal challenge, and intravenous immunization protocol for therapeutic immunotherapy against allergic asthma. E, Representative H&E staining images of lungs from PBS and nanovaccine-treated asthma mice at the end of the study. Scale bars: 500 μm (top row) and 100 μm (bottom row). F, Quantitative analysis of peribronchial inflammation in nanovaccine-treated asthma mice at the end of the study. Error bars represent mean ± SEM (n = 6 biologically independent samples). Comparisons were made using one-way ANOVA with Tukey's test. NS, no significance; *P < 0.05; **P < 0.01; ***P < 0.001.


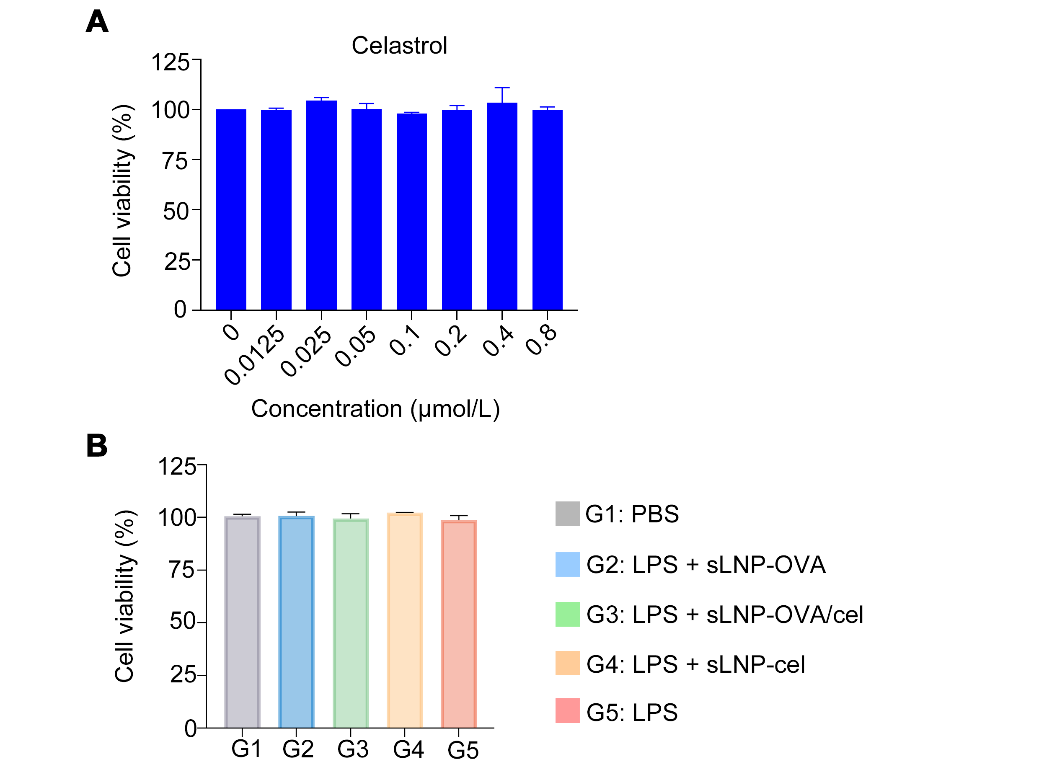


**Figure S7. Cytotoxicity of celastrol and tolerogenic mRNA-LNPs vaccine in vitro.** A, Evaluation of cytotoxicity of celastrol at different dosages on BMDCs. Error bars represent mean ± SEM (n = 3 biologically independent samples). B, Assessment of cytotoxicity of tolerogenic mRNA-LNPs vaccine on BMDCs during differentiation of tolerogenic dendritic cells from immature dendritic cells. Error bars represent mean ± SEM (n = 3 biologically independent samples).


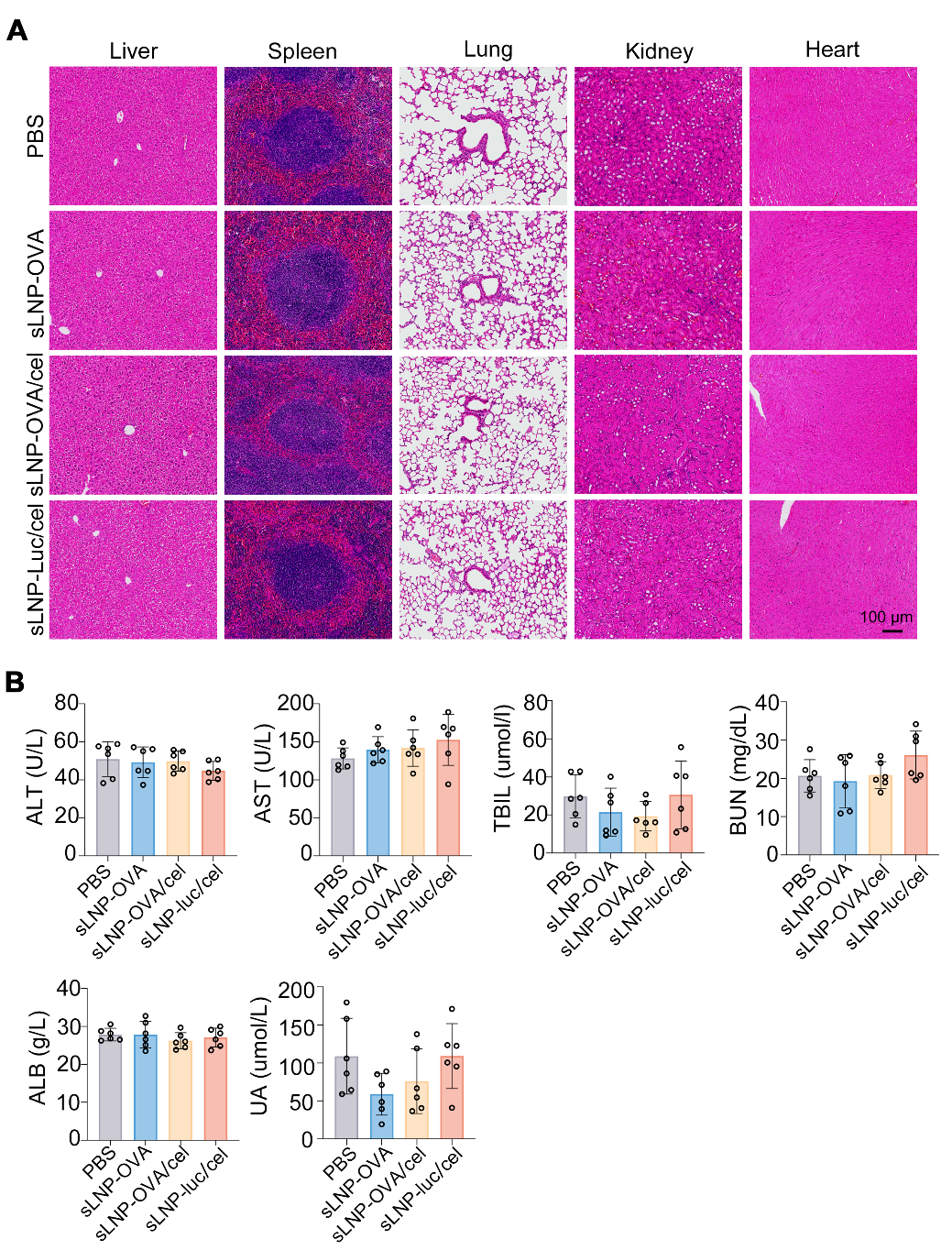


**Figure S8. Cytotoxicity of tolerogenic mRNA-LNPs vaccine in vivo.** A, Evaluation of cytotoxicity of tolerogenic mRNA-LNPs vaccine on major organs. B, Effect of immunizations on blood biochemical criteria including ALT, AST, TBIL, BUN, ALB, and UA in vivo. Error bars represent mean ± SEM (n = 6 biologically independent samples).
